# Extending the temporal window of arbovirus evolutionary analysis through the recovery of a century-old bandavirus

**DOI:** 10.1093/ve/veag034

**Published:** 2026-05-26

**Authors:** Udo Gieraths, Jörn Beheim-Schwarzbach, Matthew J Pickin, Annika Beyer, Lineke Begeman, Bernd Hoffmann, Kore Schlottau, Martin Beer, Rainer G Ulrich, Thomas Müller, Conrad M Freuling, Tiina Mauno, Marco van de Bildt, Vera C Mols, Victor M Corman, Friedemann Weber, Terry C Jones, Christian Drosten

**Affiliations:** Institute of Virology, Campus Charité Mitte, Charité - Universitätsmedizin Berlin, Corporate Member of Freie Universität Berlin and Humboldt-Universität zu Berlin, Charitépl. 1, Berlin 10117, Berlin, Germany; Faculty of Life Sciences, Humboldt Universität zu Berlin, Invalidenstraße 42, Berlin 10115, Berlin, Germany; Institute of Virology, Freiburg University Medical Center, Faculty of Medicine, University of Freiburg, Hermann Herder Straße 11, 79104 Freiburg, Baden-Württemberg, Germany; Section Clinical Virus Genomics, Institute of Virology, University Medical Center Freiburg, Hermann Herder Straße 11, 79104 Freiburg, Baden-Württemberg, Germany; Institute of Virology, Campus Charité Mitte, Charité - Universitätsmedizin Berlin, Corporate Member of Freie Universität Berlin and Humboldt-Universität zu Berlin, Charitépl. 1, Berlin 10117, Berlin, Germany; Institute for Virology, FB10-Veterinary Medicine, Justus-Liebig University, Schubertstr. 81, 35392 Gießen, Hessen, Germany; Institute of Virology, Campus Charité Mitte, Charité - Universitätsmedizin Berlin, Corporate Member of Freie Universität Berlin and Humboldt-Universität zu Berlin, Charitépl. 1, Berlin 10117, Berlin, Germany; Faculty of Life Sciences, Humboldt Universität zu Berlin, Invalidenstraße 42, Berlin 10115, Berlin, Germany; Department of Viroscience, Erasmus University Medical Centre, Dr. Molewaterplein 40, 3015GD, Rotterdam, the Netherlands; Institute of Diagnostic Virology, Friedrich-Loeffler-Institut, Federal Research Institute for Animal Health, Südufer 10, Greifswald-Insel Riems 17493, Mecklenburg-Vorpommern, Germany; Institute of Diagnostic Virology, Friedrich-Loeffler-Institut, Federal Research Institute for Animal Health, Südufer 10, Greifswald-Insel Riems 17493, Mecklenburg-Vorpommern, Germany; Institute of Diagnostic Virology, Friedrich-Loeffler-Institut, Federal Research Institute for Animal Health, Südufer 10, Greifswald-Insel Riems 17493, Mecklenburg-Vorpommern, Germany; Institute of Novel and Emerging Infectious Diseases, Friedrich-Loeffler-Institut, Federal Research Institute for Animal Health, Südufer 10, Greifswald-Insel Riems, Mecklenburg-Vorpommern, Germany; German Centre for Infection Research (DZIF), Partner Site Hamburg-Lübeck-Borstel-Riems, Südufer 10, Greifswald-Insel Riems 17493, Mecklenburg-Vorpommern, Germany; Institute for Molecular Virology and Cell Biology, Friedrich-Loeffler-Institut, Federal Research Institute for Animal Health, Südufer 10, Greifswald-Insel Riems 17493, Mecklenburg-Vorpommern, Germany; Institute for Molecular Virology and Cell Biology, Friedrich-Loeffler-Institut, Federal Research Institute for Animal Health, Südufer 10, Greifswald-Insel Riems 17493, Mecklenburg-Vorpommern, Germany; Institute of Virology, Campus Charité Mitte, Charité - Universitätsmedizin Berlin, Corporate Member of Freie Universität Berlin and Humboldt-Universität zu Berlin, Charitépl. 1, Berlin 10117, Berlin, Germany; Department of Viroscience, Erasmus University Medical Centre, Dr. Molewaterplein 40, 3015GD, Rotterdam, the Netherlands; Department of Viroscience, Erasmus University Medical Centre, Dr. Molewaterplein 40, 3015GD, Rotterdam, the Netherlands; Institute of Virology, Campus Charité Mitte, Charité - Universitätsmedizin Berlin, Corporate Member of Freie Universität Berlin and Humboldt-Universität zu Berlin, Charitépl. 1, Berlin 10117, Berlin, Germany; German Centre for Infection Research (DZIF), Partner Site Charité, Charitépl. 1, Berlin 10117, Berlin, Germany; Labor Berlin - Charité Vivantes GmbH, Sylter Straße 2, 13353 Berlin, Berlin, Germany; Institute for Virology, FB10-Veterinary Medicine, Justus-Liebig University, Schubertstr. 81, 35392 Gießen, Hessen, Germany; Institute of Virology, Campus Charité Mitte, Charité - Universitätsmedizin Berlin, Corporate Member of Freie Universität Berlin and Humboldt-Universität zu Berlin, Charitépl. 1, Berlin 10117, Berlin, Germany; German Centre for Infection Research (DZIF), Partner Site Charité, Charitépl. 1, Berlin 10117, Berlin, Germany; Centre for Pathogen Evolution, Department of Zoology, University of Cambridge, Downing Street, Cambridge CB2 3EJ, United Kingdom; Institute of Virology, Campus Charité Mitte, Charité - Universitätsmedizin Berlin, Corporate Member of Freie Universität Berlin and Humboldt-Universität zu Berlin, Charitépl. 1, Berlin 10117, Berlin, Germany; German Centre for Infection Research (DZIF), Partner Site Charité, Charitépl. 1, Berlin 10117, Berlin, Germany

**Keywords:** ancient RNA, *Bandavirus*, arbovirus evolution, bat associated viruses, temporal signal

## Abstract

Arboviruses evolve under unique ecological constraints imposed by their dual replication cycles in vertebrate and arthropod hosts. This dual-host requirement results in markedly low substitution rates, which complicate molecular clock calibration, particularly when temporal sampling spans only narrow time windows. For RNA arboviruses, wide sampling windows are especially rare due to the intrinsic instability of RNA. Here, we demonstrate that ethanol-preserved museum specimens can help overcome these temporal limitations. We successfully recovered the coding-complete genome of a bandavirus, a negative-sense segmented RNA virus that clusters with the highly pathogenic human severe fever with thrombocytopenia syndrome virus. The virus was detected in a Common pipistrelle (*Pipistrellus pipistrellus)* bat collected in northern Germany in 1919, making it one of the oldest sequenced mammalian RNA viruses, only comparable to historic measles and influenza A viruses from 1912 and 1918. Screening 1086 contemporary bat samples revealed strains of the same virus species in nine Common pipistrelle bats (2010–2018) and one Serotine bat (*Eptesicus serotinus*, 1999) from Germany and the Netherlands. Coding-complete genomes indicate frequent genome segment reassortment and widespread circulation of reassortants of this understudied virus species (*Bandavirus zwieselense*). We detected an exceptionally low substitution rate (< 6.88 × 10^−5^ substitutions/site/year) between the RdRp coding sequence of the ancient genome and its nearest modern counterpart. Additionally, functional assays demonstrated that the virus’s non-structural (NSs) protein effectively inhibits interferon induction in human HEK-293T cells. Our findings highlight the feasibility and scientific value of extracting and analysing ancient viral RNA from ethanol-preserved museum specimens to substantially enhance our understanding of RNA arbovirus evolution.

## Introduction

The evolution of RNA arboviruses is fundamentally constrained by their need to replicate in two disparate hosts (Vasilakis et al. 2009). This dual-host constraint limits strong adaptation to either host, as improved fitness in one often comes at a cost in the other (Coffey et al. 2008). Consequently, RNA arboviruses tend to evolve markedly slower than most other RNA viruses (Domingo 2020). For instance, tick-borne encephalitis virus (TBEV) exhibits substitution rates as low as 2 × 10^−5^ substitutions per site per year (s/s/y) (Bondaryuk *et al.* 2023), which is two orders of magnitude below those of rapidly evolving respiratory RNA viruses such as influenza (Patrono *et al.* 2022) or coronaviruses (Duchene *et al.* 2020a). However, detecting such low substitution rates requires sampling windows that span several decades so that a statistically significant number of substitutions can accumulate, allowing for reliable rate estimation. When the genetic distance between taxa sampled at different points in time is statistically significant, the dataset is said to exhibit a temporal signal, indicating that the population is measurably evolving (Drummond *et al.* 2003). This is an essential prerequisite for phylogenetic analyses that rely on heterochronous sampling and time-calibrated models of sequence evolution.

While a root-to-tip regression plot (Rambaut *et al.* 2016) allows a preliminary assessment of the temporal signal, only formal tests such as BETS (Duchene *et al.* 2020b) or the date-randomization test (DRT) (Duchêne *et al.* 2015) can provide the statistical validation required to confirm it. Many published arbovirus studies omit these analyses, casting serious doubt on the reliability of their reported rates. In studies that do apply them, such as work on louping ill virus with genomes from 1931 to 2015, no temporal signal was detected, despite the 84-year sampling window (Clark *et al.* 2020). Neither could a temporal signal be detected in 36 sequences of the related TBEV-EU subtype (Clark *et al.* 2020). In contrast, datasets of TBEV-FE and TBEV-common with the oldest isolate dating to 1937, have shown measurable evolution (Bondaryuk *et al.* 2023), likely reflecting differences in substitution rate, genome diversity, and sampling window. The ~ 11 kb genome of TBEV can therefore provide sufficient mutational targets for rate estimation under certain conditions, such as a very wide sampling window.

For arboviral members of the family *Phenuiviridae*, which possess much shorter tri-segmented genomes (large L, medium M, and small S genome segments) of ~ 6.4, 3.7, and 1.7 kb, even longer observation periods are required to detect a temporal signal, assuming comparable substitution rates and genomic diversity. Greater genetic diversity without increased sampling implies longer divergence times of samples since their most recent common ancestor (MRCA), increasing uncertainty in the number of accumulated substitutions and thereby weakening the temporal signal (Drummond *et al.* 2003). On the contrary, if the genetic diversity is low and a large proportion of the evolutionary history is sampled, a 20-year sampling window can be sufficient to observe a temporal signal despite relatively slow evolutionary rates (L: 1.56 × 10^−4^ s/s/y, M: 3.53 × 10^−4^ s/s/y, and S: 6.81 × 10^−4^ s/s/y), as was recently shown for severe fever with thrombocytopenia syndrome virus (SFTSV, genus *Bandavirus*, Family *Phenuiviridae,*  Pérez *et al.* 2024).

For phenuivirid species with much slower rates and a deeper history spanning centuries, only sparse sampling exists that covers a tiny fraction of its evolutionary past. Consequently, substitution rates at the lower end of the phenuivirid spectrum remain poorly characterized, a pattern that seems to affect arboviruses more generally, limiting our understanding of their origins and evolutionary dynamics.

The oldest TBEV isolates, dating to 1937, owe their existence to early Soviet research in the Russian Far East (Zlobin *et al.* 2017). Such historical isolates are exceptional, and their use is complicated by laboratory passage, which can introduce adaptation artefacts and contamination, as demonstrated in the Sofjin strain (Kovalev *et al.* 2012). To circumvent these limitations and extend temporal sampling further into the past, direct recovery of viral RNA from preserved infected tissues would be ideal. Yet, RNA’s intrinsic instability and susceptibility to RNases have historically made long-term recovery difficult.

Nevertheless, several studies have successfully retrieved decades-old viral RNA from archived medical samples, including human immunodeficiency virus 1 from 1959 (Zhu *et al.* 1998) and 1960 (Worobey *et al.* 2008), measles virus from 1912 (Düx *et al.* 2020), and influenza A virus from the 1918 pandemic (Taubenberger *et al.* 1997). Exceptional preservation in natural environments has even enabled recovery of much older RNA, such as a ~ 750-year-old barley stripe mosaic virus in grain (Smith *et al.* 2014), RNA fragments from 3400-year-old seeds (Rollo 1985), and RNA from a 14 300-year-old permafrost-preserved canid liver (Smith *et al.* 2019). Such cases are, however, restricted to environments that naturally limit RNA degradation, such as permafrost or the protective conditions within plant seeds. RNA degradation is also mitigated by human-made preservation in the form of formalin-fixed human tissues. Pathology collections hosting these tissues have been successfully used to recover ancient viral RNA (Düx *et al.* 2020, Patrono *et al.* 2022). Complementing these human samples, natural history museums house extensive collections of well-preserved animal specimens. Wet collections, which constitute a significant portion of museum specimens (Hahn *et al.* 2022), may be particularly promising for RNA virus preservation, as specimens are typically stored in 70% ethanol, an environment that limits RNA degradation *via* inactivation of RNases (Astrid 2016).

Bandaviruses (genus *Bandavirus*, family *Phenuiviridae*, order *Hareavirales*, and class *Bunyaviricetes)* are characterized by a segmented RNA genome consisting of three segments: the large (L) segment encoding the RNA-dependent RNA polymerase (RdRp), the medium (M) segment encoding the glycoprotein precursor Gn/Gc, which is post-processed into Gn and Gc, and the small (S) segment encoding the nucleocapsid (N) and non-structural (NSs) proteins. Multiple studies have demonstrated immunomodulatory functions of the bandavirus NSs protein (Wu *et al.* 2013, Ning *et al.* 2014, Santiago *et al.* 2014, Feng *et al.* 2019, Min *et al.* 2020). Among bandaviruses, SFTSV, also named Dabie bandavirus *(*species *Bandavirus dabieense)*, was identified in 2009 as the causative agent of a tick-borne disease in China (Xue-Jie *et al.* 2011). SFTSV is known to strongly suppress interferon (IFN) induction in host cells by sequestering key immune regulatory factors (e.g. interferon regulatory factor 3 and TANK-binding kinase 1) in viral inclusion bodies *via* its NSs protein (Wu *et al.* 2013, Ning *et al.* 2014, Santiago *et al.* 2014, Hong *et al.* 2019). Since its initial identification, SFTSV has been reported across Asia (Kim *et al.* 2013, Saito *et al.* 2015, Yun *et al.* 2016, Tran *et al.* 2019, Lin *et al.* 2020, Sansilapin *et al.* 2024), and related viruses such as Heartland virus (HRTV, Esguerra 2016), Guertu virus (Shen *et al.* 2018), and Kinna virus (Koka *et al.* 2024) have been discovered. Collectively, these SFTSV-like bandaviruses form a distinct clade within their genus, and reverse transcription polymerase chain reaction (RT-PCR)-based or serological evidence indicates their presence in a range of domestic and wild mammals (Niu *et al.* 2013, Hayasaka *et al.* 2016, Kimura *et al.* 2018, Matsuno *et al.* 2018, Kang *et al.* 2019, Park *et al.* 2019, Matsuu, Hamakubo, and Yabuki 2021, Kaneko *et al.* 2023), suggesting a broad host tropism. However, two additional viruses within the SFTSV-like clade have been identified exclusively in bats: Malsoor virus*,* isolated from two fruit bats, Leschenault’s rousette (*Rousettus leschenaultii*, Mourya *et al.* 2014) in India, and Zwiesel bat bandavirus (ZbbV, *species Bandavirus zwieselense*), detected in five Northern bats (*Eptesicus nilssonii*) in southern Germany (Kohl *et al.* 2020).

Here, we report the detection and genomic characterization of a century-old bandavirus recovered from an ethanol-preserved bat specimen from the wet collection of the Natural History Museum in Berlin, Germany.

## Materials and methods

### Sample collection and extraction

#### Natural History Museum

We received partial organ samples (stomach, colon, and liver) of 70 bat specimens from the Natural History Museum in Berlin (Supplementary Table S3). The specimens were preserved in 70% ethanol and, according to the curators, had not been treated with formaldehyde. Each organ sample was rinsed with Dulbecco’s phosphate-buffered saline (DPBS), cut into cubes of 1–2 mm^3^, and homogenized in DPBS using a Qiagen TissueLyser. The homogenate underwent overnight proteinase K (10 μl) digestion (10–12 h) at 56°C, followed by protease inactivation at 70°C for 10 min. After centrifugation the supernatant was transferred to a 2 ml tube and 1 ml of TRIzol™ (Invitrogen) was added, followed by standard RNA extraction according to the manufacturer’s instructions.

#### Recent bat samples, Germany

RNA extracts from pooled organs of 1000 bat specimens were obtained as described in Dafalla *et al.* (2023) and reverse transcription quantitative polymerase chain reaction (RT-qPCR) tested. For specimens that tested positive, individual organ samples (lung, liver, and colon) were homogenized in 500 μl of phosphate-buffered saline (PBS, see also Dafalla *et al.* 2023). A total of 750 μl of Qiagen AL buffer was then added to 250 μl of homogenate, followed by heat treatment at 75°C for 30 min to inactivate the potentially present virus. Subsequently, 200 μl of the inactivated homogenate was added to 700 μl Qiagen RLT buffer, and total RNA was extracted using the Qiagen RNeasy kit following the manufacturer’s protocol.

#### Recent bat samples, the Netherlands

We used carcasses from Common pipistrelle bats that were found dead, or were euthanized due to poor prognosis for survival and release, based on evaluation by bat rehabilitators in rehabilitation centres across the Netherlands. These bat carcasses were routinely frozen at −20°C until the date of transport to our facility and subsequent necropsy. Tissues from lung and liver of these bats were homogenized for 20 s in 500 μl viral transport medium using 1/4″ ceramic spheres (MP Biomedicals), followed by centrifugation at 13 000 rpm for 5 min. Subsequently, 60 μl of supernatant were added to 90 μl lysis buffer (MagNA Pure 96 External Lysis Buffer, Roche) and 3 μl Equine Arteritis virus was added as internal control. Next, RNA was isolated using an in-house developed high-throughput method (Richard *et al.* 2020). If a specimen tested positive, all available other samples of this bat were additionally tested for viral presence and viral load estimates.

### RT-qPCR primers and probe design

The following primers and a fluorescein amidite-labelled probe targeting the S segment were used: fwd: 5′-GGCTAGGTATTTAAGGATTGCTGC-3′, rev: 5′-GAAGATTGGTCGAAAGTAGCAGTG-3′, and probe: 5′-CCATGGCTTGCAGAGATTGCAGGTC-3′. Cycler conditions for RT-qPCR were as follows: 55°C for 20 min, 95°C for 3 min, 45 amplification cycles at 95°C for 15 s, 58°C for 30 s and a final cool-down step at 40°C for 30 s. SuperScript™ III RT/Platinum™ Taq Mix (Invitrogen) was used at 25 μl reaction volume according to the manufacturer’s instructions.

The extracts from the Netherlands were tested using the same primers and probe, but Fast virus 1 Step Master mix reagent was used instead: 5 μl 4xTaqman Fast virus mix; 1 μl (10 μM) fwd primer, 1 μl (10 μM) rev primer, 0.5 μl (10 μM) probe, 7.5 μl water, and 5 μl RNA (total 20 μl). Cycler conditions were as follows: 55°C for 5 min, 95°C for 20 s, 45 amplification cycles at 95°C for 3 s and at 60°C for 31 s.

### Library preparation, capturing, and sequencing

#### Ancient samples

RNA concentrations were measured using the Qubit™ RNA High Sensitivity Assay Kit (Invitrogen). Because most samples did not have detectable levels of RNA, 10 μl of undiluted extract were used for library preparation with the KAPA RNA HyperPrep Kit (Roche), following the manufacturer’s instructions. To prevent further RNA degradation, the initial fragmentation step was omitted. Libraries were sequenced to various depths on an Illumina NextSeq system.

#### Contemporary samples

A total of 100 ng of RNA was used for library preparation with the KAPA RNA HyperPrep Kit (Roche). Approximately 500 ng of each amplified library underwent viral read enrichment using the xGen™ hybridization capture kit (IDT), with capture probes consisting of interleaving 120 base pair (bp) fragments of the Zwiesel virus (MN823639–MN823641), Malsoor virus (KF186497–KF186499), and our ancient Penzlin virus (OZ243453–OZ243455) genomes. Enriched libraries were then sequenced at varying depths on an Illumina NextSeq system.

### Next-generation sequencing data processing

Sequencing data from multiple runs and organs per specimen were combined, and fastp (Chen 2023) (v0.23.2) was used to remove duplicates, filter low-quality reads, and merge overlapping paired-end reads (--merge, --dedup, --length_required 30). For virus discovery, the processed reads were aligned against a custom database of viral RNA genomes using bowtie2 (Langmead and Salzberg 2012) (v2.4.2) and the *--very-sensitive* flag. To assemble the ancient (Penzlin) virus genome, reads mapping to host ribosomal RNA were discarded (bowtie2 v2.4.2, default parameters, Langmead and Salzberg 2012), and *de novo* assembly was carried out in SPAdes (v3.15.5, default parameters, Prjibelski *et al.* 2020). For recent samples, fastp-processed reads were mapped to our assembled ancient Penzlin virus reference genome (OZ243453–OZ243455; bowtie2 v2.4.2, *--very-sensitive*, Langmead and Salzberg 2012), and consensus sequences were generated using SAMtools (v1.21, mpileup -aa -A -d 0 -Q 0, Danecek *et al.* 2021) and ivar (v1.4.3, default parameters, Grubaugh *et al.* 2019).

### Phylogenetic analysis

Multiple sequence alignments were generated using MUSCLE 5.1 (Edgar 2022) with default settings for the following datasets: (i) coding sequences (CDS) of ZbbV genomes excluding the potential mosaic strain (assembled from five different viruses) Zwiesel virus (MN823639–MN823641), (ii) L segment sequences of *Bandavirus* reference genomes (see accession numbers in Fig. 2) together with ZbbV sequences (Fig. 2), and (iii) RdRp CDS sequences of all ZbbV genomes excluding the potential mosaic strain Zwiesel virus (MN823639.1). Maximum-likelihood phylogeny: Maximum likelihood (ML) phylogenetic trees were constructed using IQ-TREE multicore version 2.3.6 (Nguyen *et al.* 2015) for MacOS Intel 64-bit. A thorough model search was performed before tree computation, and bootstrap support values were estimated using UFBoot (Hoang *et al.* 2018). For the ML tree of all bandavirus sequences (Fig. 2; Supplementary Fig. S4), additional bootstrap support values were calculated using SH-aLRT (Guindon *et al.* 2010). The resulting tree images were visualized and exported using FigTree v1.4.4 (Rambaut 2012) before final modifications were made in Inkscape v1.4 (Inkscape 2024). BEAST analysis: TempEst v1.5.3 (Rambaut *et al.* 2016) was used to confirm a temporal signal, followed by inference of dated coalescent trees using BEAST v1.10.4 (Suchard *et al.* 2018). The SRD06 substitution model was used, with base frequencies estimated from the data. An uncorrelated log-normal distributed relaxed clock (ucld) was applied, using an exponential prior with a mean rate of 0.001 substitutions per site per year. A skygrid tree prior was used. The Markov chain Monte Carlo computation was run for 80 million iterations, with sampling every 20 000 steps, and the first 8 million iterations discarded as burn-in. Convergence, mixing, and effective sample sizes (> 200 for all relevant parameters) were assessed using Tracer v1.7.2 (Rambaut *et al.* 2018). The analysis was done in two replicates, for which the results did not differ meaningfully, indicating that the chain was not stuck in a local optimum. Maximum clade credibility trees were produced using TreeAnnotator v1.10.4, summarizing the posterior distribution of trees after discarding burn-in. Final visual adjustments, including font edits and legend additions, were carried out in Inkscape v1.4 (Inkscape 2024).

### Computation of an upper limit on the substitution rate

The following analysis is based on the ML phylogenetic tree constructed from the CDS of the RdRp of the ZbbV sequences. According to this tree, the ancient sample Penzlin and the modern sample Bad Münder diverged from a common ancestor and have since accumulated 81 and 98 substitutions, respectively. We therefore infer that an additional 17 substitutions accumulated in the lineage leading to the sample Bad Münder since the sampling of Penzlin 93 years earlier. These substitutions have been estimated by the TIM2 + F + I substitution model, which was chosen by the ModelFinder algorithm of IQ-TREE 2 (Nguyen *et al.* 2015) and therefore represent an estimated genetic distance, not a simple nucleotide difference.

In order to determine an upper bound on the substitution rate it is essential to regard the number of substitutions that we observed as random. We therefore modelled the observed number of substitutions in the Penzlin (*d*_Penz_) and Bad Münder (*d*_Bad_) genomes of the RdRp CDS as realizations of Poisson-distributed random variables *X*_Penz_ and *X*_Bad_, respectively. The observed difference *δ = d*_Bad_  *− d*_Penz_ *= 17* is hence a realization of the random variable *X_δ_*, that follows a Skellam distribution.

The Skellam distribution describes the difference of two Poisson distributions, each parameterized by their expected number of substitutions (μ_Bad_ and μ_Penz_). The mean μ_Bad_ is determined by the mean of μ_Penz_ because the distance in time between the two sampling events is known and fixed to 93 years. μ_Penz_ is dependent on the unknown time in years T_Penz_ that passed since the split from the MRCA of Penzlin and Bad Münder, as well as the unknown rate *r*.


$${\mu}_{\mathrm{Penz}}=r\times{T}_{\mathrm{Penz}}\times 6255\ \mathrm{bp}$$

$${\mu}_{\mathrm{Bad}}={\mu}_{\mathrm{Penz}}+r\times 93\ \mathrm{years}\times 6255\ \mathrm{bp}$$

$${X}_{\mathrm{Penz}}= Pois\left({\mu}_{\mathrm{Penz}}\right)$$

$${X}_{\mathrm{Bad}}= Pois\left({\mu}_{\mathrm{Bad}}\right)$$

$${X}_{\delta }=\mathrm{Skellam}\left({\mu}_{\mathrm{Penz}},{\mu}_{\mathrm{Bad}}\right)$$

The aim of the derived statistical test is to calculate a *P*-value for a given substitution rate *r*, that provides the probability of observing a difference in 17 substitutions or less between the two samples: $P\left({X}_{\delta}\le 17\ |\ r\right).$

For increasing values of *r*, this *P*-value is computed and the iteration is stopped once the *P*-value drops below 5%. For each tested value of *r*, the unknown parameter value *T*_Penz_ is estimated by choosing a value that maximizes the probability to observe *X*_Bad_ *= d*_Bad_ and *X*_Penz_ *= d*_Penz_. Once *T*_Penz_ and *r* are known, the parameters μ_Penz_ and μ_Bad_ are fixed and can be used to define the Skellam distribution Skellam(*μ*_Penz*,*_  *μ*_Bad_*)*, that is needed to calculate the *P*-value.

The rate *r* that causes the *P*-value to drop below .05 is reported as the upper bound for the substitution rate.

An alternative approach to derive a threshold for the substitution rate, is to parametrize the Skellam distribution by the point estimate *μ*_Penz_ *= d*_Penz_ and *μ*_Bad_ *= d*_Bad_ given by the observed values of 81 and 98 for Penzlin and Bad Münder, respectively. The 95% quantile interval for this distribution extends up to 39 substitutions, which translates to a rate of 6.7 × 10^−5^ s/s/y.

### Writing of the manuscript

To enhance clarity, excerpts of this manuscript were rephrased using ChatGPT o1 and ChatGPT-4o.

### Plasmids

Expression constructs for the NSs proteins of SFTSV strain HB29 and ZbbV strains Penzlin and Bad Lauterberg were synthesized with N- or C-terminal 3 × FLAG tag-CDS and subcloned into expression vector pI.18 (kindly provided by Jim Robertson, National Institute for Biological Standards and Control, Hertfordshire, United Kingdom). For the two ZbbV strains, additional constructs without tags were created. The 3 × FLAG tagged HaloTag expression construct was subcloned from a plasmid purchased from Promega into pI.18 with an N-terminal 3 × FLAG tag-coding sequence. The luciferase reporter construct *p125-Luc*, expressing firefly luciferase under the control of murine *Ifnb1* promoter was kindly provided by Dr Takashi Fujita (Yoneyama *et al.* 1998), while the reporter plasmid *pRL-SV40*, constitutively expressing *Renilla* luciferase was purchased from Promega.

### Interferon induction assay

HEK-293T cells seeded at a density of 5 × 10^4^ cells per well in 24-well plates were transfected with 150 ng of *p125-luc*, which expresses firefly luciferase under the control of a *Ifnb1* promoter, alongside 50 ng of *pRL-SV40*, encoding Renilla luciferase under the control of a constitutively active promoter. In a subset of transfection mixes, pI18.MAVS-Strep, which expresses Strep-tagged mitochondrial antiviral-signalling protein (MAVS), was included to activate the *Ifnb1* promoter. Plasmids expressing the N-, C-terminally 3 × FLAG tagged or untagged NSs proteins of ZbbV or N-terminally 3 × FLAG tagged SFTSV were included in varying amounts (30, 100, and 300 ng). In parallel, an equivalent amount of N-terminally 3 × FLAG tagged HaloTag-expressing plasmid was included as a negative control. All transfections were carried out using TransIT-LT1 (Mirus Bio) with total amounts of plasmid DNA being normalized with an equivalent amount of empty pI.18 vector. After 24 h incubation the cells were processed using a dual-luciferase reporter assay system (Promega) with data read on a Berthold TriStar 2 LB942 Multimode Reader. Firefly luciferase activity was normalized to Renilla luciferase activity, and the resulting values for each construct were then expressed as a percentage of the normalized *Ifnb1* promoter activation observed with the empty vector.

### Immunofluorescence microscopy

Huh7 cells seeded on glass cover slips were transfected with plasmids expressing either the N- or C-terminally 3 × FLAG tagged recent ZbbV NSs, ancient ZbbV NSs, SFTSV NSs, N-terminally tagged HaloTag as a transfection control or left untransfected. Transfections were carried out using GeneJammer (Agilent) according to the manufacturer’s specification. After 24 h, following removal of the growth media, the cells were fixed for 30 min with 4% paraformaldehyde. Following permeabilization with 0.5% Triton X-100, the cells were blocked with bovine serum albumin and stained with the mouse anti-FLAG-M2 antibody (Sigma-Aldrich) followed by goat anti-mouse IgG Alexa Fluor 488 (Invitrogen). The coverslips were removed from the growth plate and mounted using 4′,6-diamidino-2-phenylindole-containing mounting fluid (Sigma-Aldrich). Visualization and documentation were carried out using a EVOS M5000 Imaging System (ThermoFisher).

## Results

### Defining factors of a measurably evolving Phenuiviridae population

In the following, we present the results of a simulation study that identifies the key factors enabling detection of a temporal signal in Phenuiviridae populations, underscoring the importance of long observational time spans. Following the work of Drummond *et al.* (2003), we modelled the accumulation of substitutions in an arboviral phenuivirus genome (6.4 kb, L segment) using a Poisson distribution. The mean and variance of a Poisson distribution are equal and it therefore follows, that with increased time since divergence from an MRCA of two samples, not only the expected number of accumulated substitutions increases, but also its variance. If the difference in substitutions between two samples is small, a large variance can lead to overlapping distributions that obscure a temporal signal in the data.

**Figure 1:**
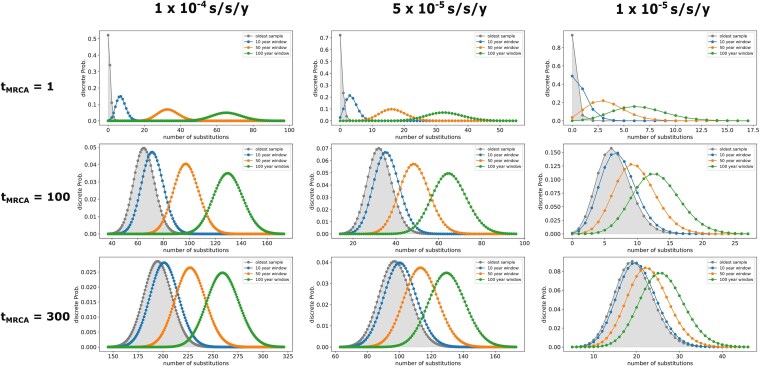
Expected accumulation of substitutions under a Poisson process for an arboviral phenuivirus genome (6.4 kb L segment) for varying substitution rates and sampling intervals. Each column represents a different substitution rate, while rows correspond to different time intervals since divergence of the oldest of two taxa from their MRCA. The x-axis shows the expected number of accumulated substitutions, calculated as the product of substitution rate, genome length, and elapsed time. The y-axis shows the corresponding discrete probability from the Poisson distribution of accumulated substitutions. The grey curve represents the distribution for the oldest sampled genome of a pair of taxa, while coloured curves indicate the distributions for genomes collected at varying temporal distances from it.

Figure 1 highlights this relationship for various choices of substitution rates and observed time windows. Each column represents a different substitution rate and each row corresponds to a different divergence time of the older sample since the MRCA of two considered samples. The x-axis denotes the expected number of accumulated substitutions, computed as the product of substitution rate, genome length, and elapsed time. The y-axis shows the discrete probability distribution of these substitution counts. The grey distribution represents the number of substitutions in the oldest sample, while coloured distributions correspond to samples collected at varying temporal distances from it. When both samples share an MRCA close to the year of sampling, the distributions of the expected number of accumulated substitutions diverge rapidly, even at the lowest rate of 1 × 10^−5^ s/s/y, within a feasible 50-year observation window.

However, in most practical cases it is not possible to sample a single viral lineage continuously over such long time periods. Randomly collected historical samples most likely trace back to MRCAs several centuries in the past. As an example, to gain a difference of 6.4 substitutions between the MRCA and a later sample, ~ 100 years are required, when assuming a rate of 1 × 10^−5^ s/s/y and a 6.4 kb genome. Consequently, for arboviruses with substitution rates in the range of 1 × 10^−5^ to 5 × 10^−5^ s/s/y, sampling windows on the order of a century or more are required to allow reliable detection of a temporal signal.

### Bandavirus presence in European bats

To evaluate whether ethanol-preserved museum specimens can extend the detectable timespan for RNA viruses, we performed RNA extraction and Illumina sequencing on stomach, liver, and colon samples from 33 bats spanning diverse species and continents (Supplementary Table S3) provided by the Natural History Museum (NHM) in Berlin, Germany. For one of the specimens, a Common pipistrelle bat from Penzlin in Germany, collected in 1919, multiple sequencing reads matched against *Malsoor* virus and ZbbV. Additional extractions from liver, lung, stomach, and colon samples of this specimen and deeper sequencing allowed us to reconstruct the coding-complete genome *via de novo* assembly.

Notably, ZbbV had not been detected in any other sequencing run performed at our institute prior to its identification in the 1919 specimen. Moreover, months later the NHM provided additional material from the same bat (further pieces of the sampled organs as well as lung tissue), and these independent extracts again contained reads matching ZbbV. In parallel, we analysed additional museum specimens (Supplementary Table S3), including 28 Common pipistrelle and nine Northern bat specimens from Germany, none of which yielded ZbbV reads. Together, these observations argue against laboratory carryover and make a widespread museum-associated contamination with ZbbV unlikely.

Since none of the additional museum samples revealed any further findings, we investigated the possible virus presence in contemporary bats to allow for comparison of recent viral genomes with our single ancient finding. To this end, we developed an RT-qPCR assay on the S segment, specific to the coding-complete ancient genome and the single available genome of ZbbV. Utilizing our RT-qPCR we screened pooled organs from 1000 samples of 20 bat species (see Supplementary Table S1) in Germany and 86 liver and lung samples from Common pipistrelle bats in the Netherlands, leading to ten positive findings. Individual organs from positive specimens were tested according to availability (Table 1; Supplementary Table S5). Positive specimens were geographically widespread across northern Germany and the Netherlands (Fig. 3a; Table 1). However, sample availability limited the tested locations, hence these findings do not necessarily provide a definitive map of virus distribution in these sampled regions.

**Table 1:** RT-qPCR positive bat specimens. The final column, *completion of CDS in percent*, shows the percentage of the genome of each coding sequence (CDS) that is covered by at least one read, when taking the ancient Penzlin virus genome as a reference. Details of sequencing breadth and coverage appear in Supplementary Table S4.

					Completion of CDS in percent			
Country	City	Year	Species	Detection in organs	L	M	NSs	N
Germany	Roßla	2011	*Pipistrellus pipistrellus*	Liver, colon	100	100	100	100
Germany	Penzlin	1919	*P. pipistrellus*	Liver, colon, lung, stomach	100	100	100	100
Netherlands	Hoogezand	2018	*P. pipistrellus*	Liver, lung, brain, colon, kidney, spleen	100	100	100	100
Germany	Gusterath	2010	*P. pipistrellus*	Liver, lung, colon	100	100	100	100
Germany	Bad Münder	2012	*P. pipistrellus*	Liver, lung, colon	100	100	100	100
Germany	Bad Lauterberg	2010	*P. pipistrellus*	Liver, lung, colon	100	100	100	100
Netherlands	Groningen	2018	*P. pipistrellus*	Lung, kidney, colon, spleen	100	96	98	99
Germany	Diekholzen	2012	*P. pipistrellus*	Brain, kidney	96	89	87	79
Germany	Sandau	1999	*Eptesicus serotinus*	Kidney	99	64	97	82
Germany	Bad Salzdetfurth	2011	*P. pipistrellus*	Lung, liver	97	39	66	95
Netherlands	Almere	2018	*P. pipistrellus*	Liver, brain	6	0	0	0

Sequencing libraries were prepared for the ten positive contemporary samples and enriched using a hybridization capture approach. Taking the ancient Penzlin virus genome as reference, for nine samples, 96%–100% of the CDS on the L-segment was covered by at least one read (breadth). The coverage varied between datasets and segments. On the L segment we saw an average coverage depth between 131 and 7721 reads across all 10 datasets. Details for the other segments with less breadth and coverage can be found in Table 1 and Supplementary Table S4. The sample ‘Almere 2018’ was excluded from further analysis, as only 6% of the genome on one segment (L) had matching reads. All sequenced viral genomes are classified under the *Bandavirus zwieselense* species, sharing over 95% amino acid sequence identity in the RdRp with the type strain Zwiesel virus (Kuhn *et al.* 2023, https://ictv.global/report/chapter/phenuiviridae/phenuiviridae/bandavirus). Only one genome of this species has been available, derived from a pooled sample of five positive bats found up to 200 km apart in Bavaria (Kohl *et al.* 2020). This consensus sequence likely reflects a mosaic of multiple viral genomes which can result in incorrect phylogenetic inferences. Moreover, within the S-segment of this type strain at least 200 nucleotides at the 5′ end of its NSs CDS are missing (Supplementary Fig. S9).

No differences in genome length were observed among the recovered sequences. GC content for each sequence is reported in Supplementary Table S4. Analysis of dN/dS ratios using BUSTED (Murrell *et al.* 2015) revealed no evidence of episodic diversifying selection. Furthermore, assessment of observed *versus* expected dinucleotide frequencies showed a marked depletion of CpG sites (Supplementary Fig. S1).

### Phylogenetic analysis of ZbbV genomes

To broadly contextualize our sequences within the *Bandavirus* genus, we constructed an ML phylogenetic tree of the L segment nucleotide sequences using representative genomes from all known *Bandavirus* species, as well as the two unclassified Malsoor virus genomes.

**Figure 2:**
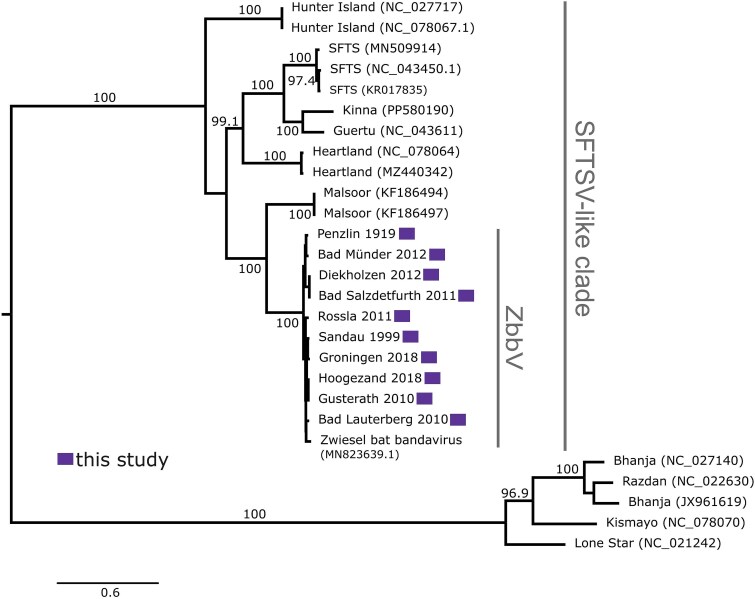
Phylogenetic relationships of strains of the *Bandavirus* genus on the L segment. The maximum-likelihood tree was computed using IQ-TREE 2.3.6 (Nguyen *et al.* 2015). Rift Valley fever virus (NC_014397.1) was used as an outgroup for rooting and subsequently removed for plotting. Bootstrap support values were computed using UFBoot (Hoang *et al.* 2018) and only displayed if the support is ≥ 95%. Within the *ZbbV* clade no bootstrap support values are plotted for visual conciseness. Supplementary Fig. S4 shows the raw tree output, including the outgroup and UFBoot (Hoang *et al.* 2018) and SH-aLRT (Guindon *et al.* 2010) bootstrap values for all branches.

All ZbbV genomes of the L segment cluster within the SFTSV-like bandavirus clade with 100% bootstrap support (Fig. 2). In this broader SFTSV-like clade, Malsoor virus and ZbbV together constitute a distinct lineage, identified thus far exclusively in bats. Moreover, the ZbbV sequences, encompassing the ancient (Penzlin 1919) genome, constitute a separate well-supported subclade (100% bootstrap support) distinct from Malsoor virus.

Focusing exclusively on our recovered ZbbV genomes, we computed ML trees for each CDS using MUSCLE 5.1 (Edgar 2022) and IQ-TREE (Nguyen *et al.* 2015). The phylogenetic analysis revealed two well-supported clades, hereafter referred to as clades A and B, which were consistently observed across all four CDSs. Furthermore, two sequences with minimal genetic divergence, collected 1 year and 9 km apart (Diekholzen and Bad Salzdetfurth), stand out by forming a subclade with a long branch length in each CDS tree. By contrast, two samples from the Netherlands (Groningen and Hoogezand), both collected in 2018 and located 16 km apart, do not occur in a distinct subclade; in fact, except for the RdRp tree, they appeared in different clades across all other CDS trees. Due to the limited number of samples and short coding regions, the support for deeper clades in the phylogeny is low, particularly for the NSs and N CDS on the S segment.

**Figure 3:**
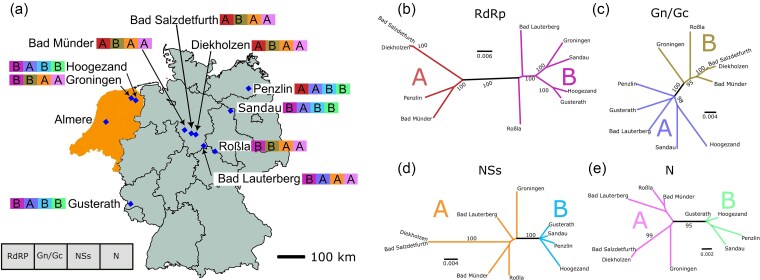
Locations of virus detection and phylogenetic relationships. (a) Map indicating the location of positive tested bats as well as their colour-coded composition of CDSs from the two major clades. Unrooted phylogenetic ML trees of the CDS of (b) RdRp, (c) Gn/Gc, (d) NSs, and (e) N. Each of the ML trees is colour coded according to the two major clades A and B. The colour coding highlights the composition of each viral genome in (A). The raw unmodified trees can be found in Supplementary Figs S5–S8.

Panels B–E of Fig. 3 present the ML phylogenetic trees for each CDS (RdRp, Gn/Gc, NSs, and N), with clades A and B highlighted. The samples in the map (Fig. 3a) carry a colour code indicating their clade membership. All samples exhibit an identical clade membership regarding the two CDSs on the S segment (NSs:A, N:A or NSs:B, N:B). Across the 10 samples, five distinct clade membership combinations across the segments were detected (Fig. 3), suggesting multiple reassortment patterns.

The samples from Diekholzen, Bad Salzdetfuth, and Bad Münder share a common reassortment pattern (RdRp: clade A, Gn/Gc: clade B, NSs: clade A, N: clade A) and are geographically in close proximity (Fig. 3). Despite being separated by several hundred kilometres, the samples from Hoogezand, Sandau, and Gusterath also share a common reassortment pattern (RdRp: clade B, Gn/Gc: clade A, NSs: clade B, and N: clade B). A third pattern (RdRp: clade B, Gn/Gc: clade B, NSs: clade A, and N: clade A) is found for the samples from Groningen and Roßla that were collected hundreds of kilometres apart. Finally, the ancient sample Penzlin and the sample Bad Lauterberg possess a unique pattern of clade membership (Fig. 3).

### Functional characterization of the NSs protein

Seeking additional confirmation that the assembled genomes represent an authentic virus, we performed functional assays. The ZbbV NSs protein was chosen as the NSs proteins of bandaviruses have a well-established role in antagonizing IFN induction (Wu *et al.* 2013, Ning *et al.* 2014, Santiago *et al.* 2014, Feng *et al.* 2019, Min *et al.* 2020), a functionality we considered likely to be conserved for the ZbbV NSs protein. As an NSs protein, the NSs protein functions largely independently of other viral proteins, making it a suitable candidate for functional characterization. To explore the functional properties of the ZbbV NSs protein, we examined its ability to suppress IFN induction and promote the formation of inclusion bodies.

To assess whether the ZbbV NSs protein can inhibit *Ifnb1* promoter activation, luciferase reporter assays were carried out in HEK-293T cells. The reporter plasmids expressing luciferase under the control of the *Ifnb1* promoter were co-transfected with plasmids expressing MAVS, the key adapter protein mediating IFN induction in response to RNA viruses. Additionally, varying amounts of plasmids expressing the NSs proteins from the Penzlin 1919 sequence (ancient), Bad Lauterberg 2010 sequence (recent), SFTSV or a negative control were co-transfected to compare their functionality. We were unable to compare with the type strain Zwiesel virus, since its NSs gene is incomplete (Supplementary Fig. S9).

**Figure 4:**
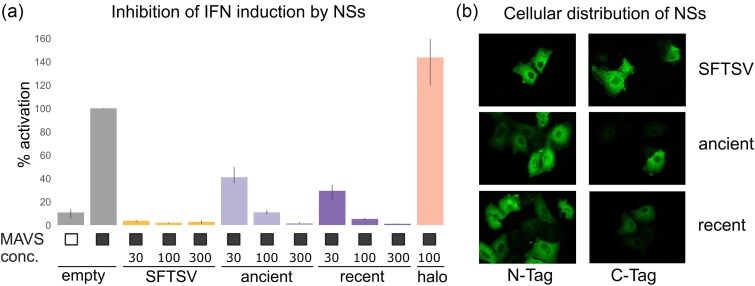
Functional characterization of the NSs protein (a) firefly luciferase assay to measure *Ifnb1* promoter activation *via* MAVS overexpression in HEK 293 T cells. Black empty or filled squares indicate whether MAVS was overexpressed or not. Below these, concentrations (conc.) of the respective plasmids are given in nanograms. Firefly luciferase activity was normalized to Renilla luciferase. The percentage of the activation of the empty vector is given for N-terminally 3 × FLAG tagged SFTSV NSs, untagged Penzlin 1919 (ancient) NSs, untagged bad Lauterberg 2010 NSs (recent) and N-terminally 3 × FLAG tagged HaloTag as additional negative control. Error bars indicate estimated 95% confidence intervals. (b) Huh7 cells were transfected with plasmids expressing either the N- or C-terminally 3 × FLAG tagged bad Lauterberg 2010 NSs (recent), Penzlin 1919 NSs (ancient) or SFTSV NSs and stained with mouse anti-FLAG antibody followed by goat anti-mouse IgG Alexa fluor 488.

Both the ancient and recent NSs proteins demonstrated a strong, dose-dependent inhibition of MAVS-induced *Ifnb1* promoter activation (Fig. 4a). At the two highest amounts of plasmid, the inhibitory effect of both the ancient and recent untagged NSs is comparable to that of the human pathogenic SFTSV N-tagged NSs protein that was used as control. No relevant difference was observed between the inhibitory activity of the ancient and recent NSs protein variants.

To assess the expression levels and cellular distribution of the ZbbV NSs protein, we expressed N- and C-terminally 3 × FLAG tagged ZbbV NSs proteins in Huh7 cells using both the ancient and recent variants. The cellular distribution of the NSs proteins was assessed using fluorophore-conjugated anti-FLAG antibody staining. Cells transfected with plasmids expressing tagged SFTSV NSs protein were used as a positive control. As expected, the positive controls produced distinct inclusion bodies in the cells consistent with previous descriptions (Wu *et al.* 2013, Ning *et al.* 2014, Santiago *et al.* 2014, Hong *et al.* 2019) (Fig. 4b). In contrast, neither the ancient nor recent ZbbV NSs variants formed inclusion bodies. Instead, the NSs protein was evenly distributed throughout the cells, showing no evidence of localized concentration for the N- or the C-tagged ZbbV NSs proteins. Nevertheless, both tagged proteins retain their IFN induction inhibitory activity in HEK-293T cells (Supplementary Figs S2 and S3).

### Substitution rate of ZbbV

**Figure 5:**
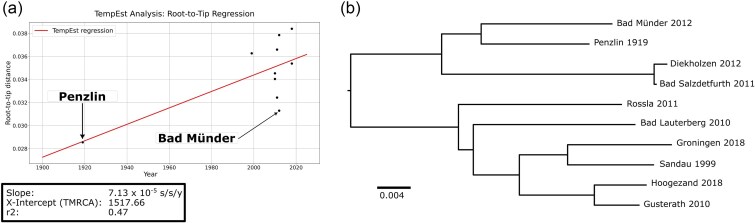
TempEst regression and phylogenetic tree calculated on the RdRp CDS of our ZbbV genomes with the ‘heuristic residual mean squared’ rooting method. (a) Scatter plot of root-to-tip distances *versus* sampling year. The computed regression line from TempEst is shown in red. TMRCA: time of the most recent common ancestor; r2: coefficient of determination. (b) Maximum-likelihood phylogenetic tree computed using IQ-TREE and rooted by TempEst, with sample name and date of sampling specified at the tip nodes.

An initial assessment of our ZbbV genomes for a temporal signal on the RdRp CDS that largely covers the L segment was obtained using TempEst v1.5.3 (Rambaut *et al.* 2016). Given the substantially shorter lengths (and thus reduced information content) of the other CDSs, we primarily focused on the longest CDS of the RdRp. The resultant root-to-tip distance plot (Fig. 5a) shows an upward slope of the inferred regression line with a rate of 7.13 × 10^−5^ s/s/y and a coefficient of determination (*r*^2^) of 0.47. While the positive slope indicates a temporal signal, the coefficient of determination hints that the data do not conform to a strict molecular clock model. This can also be seen in the phylogenetic tree obtained using the ‘heuristic residual mean squared’ rooting method of TempEst (Fig. 5b), which was the basis for the computation of the root-to-tip regression line. Several root-to-tip distances of samples exhibit inconsistencies with their sampling dates under the strict molecular clock assumption. The most pronounced example is the sample Sandau 1999 which was sampled 12 years prior to Rossla 2011, but displays a larger root-to-tip distance. However, the century-old sample Penzlin 1919 exhibits the shortest root-to-tip distance, and its inclusion reveals a clear temporal signal despite the substantial variation observed among the more recent samples.

Given this prior insight into temporal signal and clock-rate, we inferred a dated coalescent Bayesian phylogeny on the RdRp CDS of our ZbbV genomes with a relaxed molecular clock using BEAST v1.10.4 (Suchard *et al.* 2018). The inferred Bayesian parameter estimates exhibit large uncertainties, due to the limited number of informative samples (Supplementary Fig. S10). As the sampling intervals between the contemporary samples are most likely too short to confer a temporal signal, only measurements between contemporary and the single ancient sample allow for informative rate estimates. Hence, the large space of per-branch rate combinations couldn’t reasonably be constrained by the limited data, resulting in many equally plausible configurations that inflated the uncertainty of the estimates. This high uncertainty in the parameter estimates explains the failure to confirm a temporal signal using the DRT (Duchêne *et al.* 2015) (Supplementary Fig. S11). Furthermore, across repeated runs we encountered numerical underflow leading to unstable and non-interpretable marginal likelihood estimates, when attempting to perform a BETS analysis. It was therefore not possible to report rate estimates with certainty using the dated Bayesian phylogeny.

**Figure 6:**
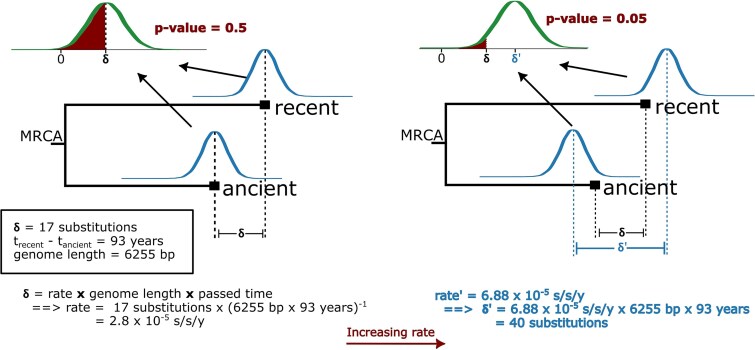
Illustration of the computation of an upper limit on the substitution rate between the Penzlin (ancient) and bad Münder (recent) sample. On the left side a point estimate (*n* = 1) of the mean number of substitutions is made for each taxa, to model their distribution by a Poisson distribution. The difference in accumulated substitutions (δ) between the ancient and recent sampling is described by a Skellam distribution, which allows computation of a *P*-value for the observed substitutions. Increasing the rate leads to a larger δ, again modelled by a Skellam distribution. With increasing rates, the distribution for the expected number of substitutions will assign a very low probability to the actual observed number of substitutions, as quantified by a *P*-value. The rate that causes a *P*-value below .05 represents the upper threshold on the substitution rate that could reasonably account for the observed data within the clade encompassing the ancient and recent samples.

To nevertheless leverage the unique value of the generated dataset, we focused on the ancient sample and its genetically closest counterpart on the RdRp CDS to determine an upper threshold on the substitution rate (Fig. 6). These two genomes, sampled 93 years apart, have 97% nucleotide identity on the RdRp CDS, corresponding to an estimated 17 additional substitutions that accumulated in the modern sample since the sampling of the ancient specimen. This genetic distance of 17 substitutions is taken from an ML-derived phylogenetic tree computed with IQ-TREE 2.3.6 (Nguyen *et al.* 2015) using the TIM2 + F + I substitution model. To assess whether this level of divergence is compatible with a given substitution rate, we developed a statistical test to compute a *P*-value that provides the probability to observe 17 substitutions or less in a time span of 93 years, assuming a given substitution rate. Using this approach we calculated that only a rate of less than 6.88 × 10^−5^ s/s/y could most likely explain the observed substitutions that accumulated over the sampling window (*P*-value: .047). While the choice of the common 5% threshold is somewhat arbitrary, it serves as a guideline for identifying a range of substitution rates that could reasonably account for the observed data within the clade encompassing the samples Bad Münder and Penzlin.

This upper threshold is only valid within the considered sub-clade. We can observe its violation by the informally derived rate from TempEst modelling the entire species with a strict clock rate. While the actual observed rate between the two samples Bad Münder and Penzlin is 2.8 × 10^−5^ s/s/y (Fig. 6, left), the upper threshold covers the possibility that the observed data are extreme samples from a distribution with a much higher substitution rate (Fig. 6, right). The rate of 6.88 × 10^−5^ s/s/y thus likely represents a limit that applies to the RdRp CDS of all yet undiscovered genomes in the clade containing these two samples.

## Discussion

The presented work highlights the potential of museum collections to contribute to RNA virus evolution studies. We report the successful extraction of viral RNA from an ethanol-preserved specimen after 100 years of storage, establishing that such museum specimens can preserve viral RNA of sufficient integrity to enable phylogenetic and functional analyses. The recovered coding-complete viral genome enabled follow-up studies on virus presence in contemporary samples and provided a means to estimate an upper limit on the substitution rate over a century-long timescale, despite the relatively short genome length. Functional analysis of one of the encoded virus proteins validated the integrity of the assembled sequence and reinforced confidence in the authenticity of all the recovered viral genomes.

Our work extends on the findings of the first five ZbbV strains in Bavaria previously described by Kohl *et al.* (2020), by reporting additional findings in midwest and northern Germany as well as the Netherlands. Furthermore, we identified two additional bat species (*Pipistrellus pipistrellus, Eptesicus serotinus*) susceptible to infection and sequenced six coding-complete genomes and four partially complete genomes. Infection appears to be widespread across multiple organs, with viral RNA detected in liver, lung, faeces, colon, brain, kidney, and spleen. Given that the related SFTSV is suspected to be primarily transmitted *via* ticks (Luo *et al.* 2015, Jang *et al.* 2024), a similar transmission route for ZbbV appears plausible. The absence of evidence for episodic diversifying selection is consistent with expectations for arboviruses, whose evolution is typically dominated by purifying selection due to their dual-host life cycle. The pronounced depletion of CpG dinucleotides suggests past adaptation of this viral species to mammalian hosts (Supplementary Fig. S1).

The 10 genome sequences we generated allowed the first phylogenetic analysis of the *Bandavirus zwieselense* species. Clade membership appears independent of sampling location and time (Fig. 3), indicating substantial undersampling. As additional genomes are discovered, we expect more subclades to emerge that link clade membership with temporal and geographic patterns, as seen with samples from Diekholzen and Bad Salzdetfurth.

The clear division into two well-supported clades per segment led us to visualize putative reassortment patterns, hinting at frequent reassortment events within this species. Notably, these patterns are geographically and temporally widespread, suggesting co-existing reassortants.

Without virus isolates, any *de novo* assembly method carries the risk of generating chimeric sequences by incorrectly combining viral and non-viral fragments (e.g. from the host or associated microbes). The low sample quality did not allow for virus isolation and no readily available reverse genetics system exists. Hence, we employed the described overexpression approach to characterize one of the viral proteins and additionally validate the accuracy of our assembled genome. Our findings confirmed that the NSs proteins of both ZbbV strains are functional, exhibiting clear anti-IFN induction activity. A strong dose-dependent inhibitory effect on IFN induction was observed, a feature that is known in SFTSV and HRTV, two human pathogenic bandaviruses (Wu *et al.* 2013, Ning *et al.* 2014, 2017, Hong *et al.* 2019). This inhibitory effect was observed in a human kidney cell line, suggesting that the host immune components targeted by the ZbbV NSs protein are likely conserved across hosts and that its IFN inhibition mechanism may extend to a broader range of species. Although the exact inhibitory process remains to be determined, the absence of inclusion bodies in fluorescently labelled ZbbV NSs-expressing cells (Fig. 4b), where N- and C-terminally tagged NSs proteins retained their inhibitory effect (Supplementary Figs S2 and S3), implies that the mechanism of IFN induction inhibition is distinct from that of SFTSV.

An initial substitution rate analysis using TempEst identified a temporal signal and suggested a preliminary average rate of 7.13 × 10^−5^ s/s/y on the RdRp CDS. However, it also became apparent that substitution rates vary between clades and therefore prohibit the use of the strict molecular clock model. Due to the limited number of samples, only one or two genomes can guide the computation of the individual rates of a relaxed clock model, making the estimates from the dated Bayesian phylogeny on the RdRp CDS highly uncertain. This uncertainty led to the failure of the DRT, thereby invalidating the derived estimates. To generate a reliable dated phylogeny with confident clade-specific rate estimates, additional historical genomes will therefore be required. Although the other historical samples tested in this study were negative (Supplementary Table S3), the successful recovery of genomic material from a century-old specimen demonstrates the general feasibility.

To nevertheless obtain a robust rate estimate under current data constraints, we focused solely on the RdRp CDS of the ancient genome and its closest modern counterpart, Bad Münder. We developed a statistical test to determine an upper threshold on the substitution rate that could reasonably account for the divergence between the two CDSs. The resulting upper bound of 6.88 × 10^−5^ s/s/y provides a conservative, statistically solid estimate of an observed arboviral RNA substitution rate across a century. Although the actual measured rate between these CDSs (Penzlin and Bad Münder) is lower, the derived upper bound likely represents a limit that applies to the RdRp CDS of all undiscovered genomes in the clade encompassing both genomes.

This rate is considerably lower than that reported for the related SFTSV bandavirus on the L segment that is largely covered by the RdRp CDS (mean rate 1.56 × 10^−4^ s/s/y, Pérez *et al.* 2024). It is important to note that the reported SFTSV rate represents a mean across all clades, whereas the upper limit calculated here relates to a single subclade. Moreover, Pérez *et al.* proposed a staggered timeline of emergence for SFTSV segments, with the S segment added last with an estimated MRCA around 1985 and increasing genetic diversity across L and M segments appearing from the 1970s onwards (Pérez *et al.* 2024). In contrast, ZbbV likely follows a different evolutionary trajectory. The close genetic similarity between the 1919 and 2012 genome on the L segment argues against a recent expansion in diversity, as such low divergence over nearly a century would be incompatible with a recent, rapid diversification process.

The other closely related bandavirus HRTV likewise exhibits substantially higher substitution rates (S segment: 5.3 × 10^−4^ s/s/y) and a MRCA of all available genomes of the S segment dating within the past 75 years (Bellman *et al.* 2025). These findings suggest that SFTSV and HRTV emerged relatively recently, within the past century. In contrast, our analysis of the ZbbV subclade containing the Penzlin and Bad Münder samples indicates an MRCA predating the year 1750 when applying the inferred upper bound on the substitution rate. We were unable to perform equivalent temporal analyses for the other CDSs or segments, and therefore cannot assess whether ZbbV exhibits segment-specific (staggered) emergence dynamics similar to those reported for SFTSV. Nonetheless, the origin of the ZbbV species likely extends much further back in time than for SFTSV or HRTV, implying that *Bandavirus zwieselense* represents an older, more slowly evolving species within the *Bandavirus* genus. The deeper evolutionary origin raises the possibility that ancestral bandaviruses related to this species contributed one or more segments to the reassortment events, which gave rise to contemporary SFTSV (Pérez *et al.* 2024). The stark difference in substitution rate might also be attributable to the time-dependent rate phenomenon, which states that molecular evolution rates can appear to vary, depending on the timescale over which they are measured (Duchêne *et al.* 2014, Aiewsakun *et al.* 2016). The upper bound represents a rate measured over a long-term (~100 years) during which purifying selection removed many slightly deleterious mutations and saturation might already be reached for some positions.

In contrast, for SFTSV and HRTV the rates are estimated over a time frame of ~ 20 years, representing more recently emerging bandaviruses and display comparatively elevated substitution rates, that may still be in flux and can therefore not be directly compared to ZbbV. ZbbV has maintained low substitution rates over the last century, similar to TBEV, with values well below 10^−4^ s/s/y. This similarity highlights a potential link between vector ecology and evolutionary rate. Both TBEV and ZbbV are presumably tick-borne, whereas mosquito-borne arboviruses generally evolve faster, with substitution rates exceeding 10^−4^ s/s/y. The lower apparent rates observed in tick-borne viruses may reflect the infrequent feeding and transmission cycles of their vectors, which limit opportunities for mutation fixation. Nonetheless, additional historical genomes from a broader range of arboviruses will be required to rigorously test this hypothesis. As illustrated by SFTSV and HRTV, potential effects of vector ecology on inferred evolutionary rates may be difficult to resolve when estimates rely on short sampling windows (~20 years). The availability of longer time-calibrated estimates as they exist for the mosquito transmitted Dengue virus (~60 years, Jagtap *et al.* 2023) and for the tick-transmitted TBEV/ZbbV (~90 years) makes differences between mosquito- and tick-associated viruses more apparent, underscoring the value of historical genomes for detecting subtle, long-term effects that are unlikely to be discernible in modern datasets.

In future research, an unbiased sampling of contemporary bat and tick populations, coupled with serological surveys, could offer deeper insights into the distribution of ZbbV in Europe. Our demonstration of the feasibility of sequencing ancient ethanol-preserved viral RNA genomes from museum samples may encourage further investigations, ultimately refining molecular clock rate estimates. Finally, the presented functional work raises the question of the precise mechanism that allows the NSs protein to inhibit IFN induction, a subject that should also be investigated in bat cell lines to understand host-specific differences and assess the virus’ zoonotic potential.

## Study limitations

While mean sequencing depth is high for most genomes (Supplementary Table S4), local coverage varies, and positions with very low depth are intrinsically more sensitive to stochastic Illumina sequencing errors (reported at ~ 0.5% for Illumina NextSeq). Accordingly, occasional miscalls in the consensus sequence cannot be excluded at isolated low-coverage sites (including single-read positions). However, because such positions are infrequent, any resulting errors are expected to have a negligible impact on downstream analyses and resultant conclusions.

## Supplementary Material

VirusEvolution_gieraths_revised_manuscript_supp_clean_veag034

## Data Availability

The sequencing data for this study have been deposited in the European Nucleotide Archive (ENA) at EMBL-EBI under accession number PRJEB86147 and will be made publicly available upon publication. All code to reproduce the findings, figures, and tables is available at the following URL: https://github.com/UdoGi/ancient_bunyavirus.
