# Supplementary material for: Extending the temporal window of arbovirus evolutionary analysis through the recovery of a century-old bandavirus
Source: Virus Evol. 2026 May 26;12(1):veag034. doi: 10.1093/ve/veag034 (PMC13317966; doi:10.1093/ve/veag034)
Supplement: VirusEvolution_gieraths_revised_manuscript_supp_clean_veag034 [file virusevolution_gieraths_revised_manuscript_supp_clean_veag034.docx]

# Supplementary Materials

## Supplementary Tables

**Supplementary Table 1:**

Origin of bat specimens by federal states in Germany

| **Federal state** | **# of specimens** |
| --- | --- |
| Lower Saxony | 248 |
| Berlin | 204 |
| Saxony-Anhalt | 183 |
| Baden-Wuerttemberg | 118 |
| unknown | 85 |
| Mecklenburg-Western Pomerania | 59 |
| Rhineland-Palatinate | 26 |
| Brandenburg | 25 |
| Saarland | 21 |
| Hesse | 15 |
| Schleswig-Holstein | 7 |
| North Rhine-Westphalia | 5 |
| Thuringia | 4 |

**Supplementary Table 2:**

Species composition of sampled contemporary bats in Germany

| **Species** | **# of specimens** | **# tested postive** |
| --- | --- | --- |
| *Pipistrellus pipistrellus* | 355 | 6 |
| *Nyctalus noctula* | 177 | 0 |
| Not determined | 73 | 0 |
| *Eptesicus serotinus* | 51 | 1 |
| *Myotis nattereri* | 49 | 0 |
| *Myotis mystacinus* | 44 | 0 |
| *Myotis daubentonii* | 40 | 0 |
| *Plecotus auritus* | 39 | 0 |
| *Pipistrellus nathusii* | 30 | 0 |
| *Pipistrellus* spec*.* | 24 | 0 |
| *Vespertilio murinus* | 23 | 0 |
| *Nyctalus leisleri* | 18 | 0 |
| *Myotis myotis* | 17 | 0 |
| *Myotis brandtii* | 12 | 0 |
| *Plecotus austriacus* | 12 | 0 |
| *Pipistrellus pygmaeus* | 12 | 0 |
| *Myotis bechsteinii* | 6 | 0 |
| *Myotis myst/ bra /alca* | 5 | 0 |
| *Barbastella barbastellus* | 4 | 0 |
| *Myotis dasycneme* | 3 | 0 |
| *Eptesicus nilssonii* | 2 | 0 |
| *Myotis* spec*.* | 2 | 0 |
| *Plecotus* spec*.* | 1 | 0 |
| *Pipistrellus kuhlii* | 1 | 0 |

**Supplementary Table 3:**

Composition of species and countries of origin of the samples from the Natural History Museum in Berlin. Two rounds of sampling took place. The first round covered various continents and species. The second targeted only Germany and the two bat species known to have been infected by ZbbV.

| **Sampling round** | **Country** | **Family** | **Genus** | **Species** | **# of specimens** | **Min year** | **Max year** |
| --- | --- | --- | --- | --- | --- | --- | --- |
| 1 | Cameroon | Pteropodidae | *Eidolon* | *helvum* | 6 | 1905 | 1937 |
| 1 | Cyprus | Pteropodidae | *Rousettus* | *aegyptiacus* | 3 | 1912 | 1912 |
| 1 | Germany | Vespertilionidae | *Pipistrellus* | *pipistrellus* | 8 | 1816 | 2000 |
| 1 | Indonesia | Molossidae | *Chaerephon* | *plicatus* | 2 | 1899 | 1926 |
| 1 | Israel | Pteropodidae | *Rousettus* | *aegyptiacus* | 5 | 1910 | 1910 |
| 1 | Italy | Vespertilionidae | *Nyctalus* | *noctula* | 1 | 1908 | 1908 |
| 1 | Madagascar | Pteropodidae | *Eidolon* | *helvum* | 1 | 1907 | 1907 |
| 1 | Peru | Phyllostomidae | *Desmodus* | *rotundus* | 1 | 1911 | 1911 |
| 1 | Poland | Vespertilionidae | *Nyctalus* | *noctula* | 2 | 1915 | 1919 |
| 1 | Rwanda | Pteropodidae | *Eidolon* | *helvum* | 3 | 1908 | 1908 |
| 1 | Switzerland | Vespertilionidae | *Nyctalus* | *noctula* | 1 | 1907 | 1907 |
| 2 | Germany | Vespertilionidae | *Eptesicus* | *nilssonii* | 9 | 1982 | 1986 |
| 2 | Germany | Vespertilionidae | *Pipistrellus* | *pipistrellus* | 28 | 1902 | 2000 |

**Supplementary Table 4:**

Sequencing details for positive tested specimens. The sequencing reads across all organs of a specimen were merged for the purpose of genome assembly. Breadth: percentage of the genome covered by at least one read.

| **seg** | **sample** | **min coverage** | **max coverage** | **total mapped reads** | **mean coverage** | **breadth** | **% GC** |
| --- | --- | --- | --- | --- | --- | --- | --- |
| L | Bad Lauterberg 2010 | 50 | 8,044 | 2,041,742 | 7721.74 | 100.00 | 43.09 |
| L | Bad Münder 2012 | 300 | 8,032 | 681,046 | 7475.53 | 100.00 | 42.94 |
| L | Bad Salzdetfurth 2011 | 1 | 4,226 | 26,104 | 568.19 | 97.57 | 43.19 |
| L | Diekholzen 2012 | 2 | 2,175 | 17,530 | 325.90 | 94.14 | 42.90 |
| L | Groningen 2018 | 1 | 424 | 5,362 | 131.77 | 99.95 | 42.92 |
| L | Gusterath 2010 | 4 | 4,665 | 77,135 | 1629.64 | 99.86 | 42.92 |
| L | Hoogezand 2018 | 44 | 8,008 | 203,488 | 4800.49 | 100.00 | 42.94 |
| L | Penzlin 1919 | 2 | 432 | 10,447 | 203.97 | 100.00 | 42.87 |
| L | Rossla 2011 | 286 | 6,226 | 96,238 | 2573.23 | 100.00 | 42.82 |
| L | Sandau 1999 | 1 | 2,517 | 13,095 | 245.43 | 90.76 | 43.12 |
| M | Bad Lauterberg 2010 | 30 | 8,043 | 1,617,592 | 7540.02 | 100.00 | 45.66 |
| M | Bad Münder 2012 | 39 | 8,072 | 1,592,158 | 7584.81 | 100.00 | 46.02 |
| M | Bad Salzdetfurth 2011 | 5 | 1,020 | 2,576 | 82.29 | 36.61 | 46.67 |
| M | Diekholzen 2012 | 1 | 1,613 | 5,855 | 205.92 | 84.72 | 46.76 |
| M | Groningen 2018 | 1 | 572 | 3,249 | 132.42 | 95.24 | 45.99 |
| M | Gusterath 2010 | 2 | 8,000 | 78,955 | 3053.95 | 99.94 | 46.18 |
| M | Hoogezand 2018 | 5 | 1,698 | 3,805 | 187.15 | 99.71 | 45.96 |
| M | Penzlin 1919 | 1 | 2,098 | 31,950 | 1162.82 | 100.00 | 45.83 |
| M | Rossla 2011 | 24 | 8,004 | 118,702 | 4931.34 | 99.97 | 45.68 |
| M | Sandau 1999 | 1 | 1,177 | 2,270 | 74.65 | 62.75 | 46.65 |
| S | Bad Lauterberg 2010 | 160 | 8,057 | 665,167 | 7386.96 | 100.00 | 45.44 |
| S | Bad Münder 2012 | 88 | 8,055 | 459,195 | 7072.56 | 100.00 | 45.15 |
| S | Bad Salzdetfurth 2011 | 1 | 860 | 2,703 | 229.01 | 80.59 | 45.91 |
| S | Diekholzen 2012 | 1 | 993 | 2,482 | 172.47 | 85.18 | 45.44 |
| S | Groningen 2018 | 2 | 395 | 1,270 | 105.45 | 95.24 | 45.64 |
| S | Gusterath 2010 | 2 | 8,023 | 107,678 | 4902.16 | 100.00 | 45.44 |
| S | Hoogezand 2018 | 1 | 8,018 | 34,508 | 2906.59 | 100.00 | 45.41 |
| S | Penzlin 1919 | 1 | 701 | 5,896 | 423.06 | 100.00 | 45.29 |
| S | Rossla 2011 | 37 | 8,004 | 36,603 | 3476.72 | 100.00 | 45.38 |
| S | Sandau 1999 | 3 | 1,572 | 4,599 | 308.89 | 88.26 | 45.89 |

**Supplementary Table 5:**

RT-qPCR details on positive tested specimens. The detected copies per microliter (cp/µl) are shown for each positive tested organ. neg: tested negative, na: sample not available.

|  | Almere 2018 | Hoogezand 2018 | Groningen 2018 | Gusterath 2010 | Diekholzen 2012 | Bad Salzdetfurth 2011 | Bad Münder 2012 | Roßla 2011 | Sandau 1999 | Bad Lauterberg 2010 |
| --- | --- | --- | --- | --- | --- | --- | --- | --- | --- | --- |
| Liver | 1.7e0 | 1e2 | neg | 9.5e2 | neg | 1.1e2 | 1.7e3 | 1e1 | neg | 5.3e2 |
| Lung | neg | 3.7e2 | 2.3e2 | 7e2 | 2.7e0 | 2.1e2 | 8.5e2 | neg | neg | 1.1e3 |
| Feces | neg | 9.3e0 | neg | na | na | na | na | na | na | na |
| Nose wash | neg | neg | neg | na | na | na | na | na | na | na |
| Colon | neg | 3.7e2 | 4.9e1 | 2.4e1 | neg | neg | 1.1e1 | 7.7e1 | neg | 8.2e0 |
| Brain | 5.1e0 | 1.7e1 | neg | na | 6.7e1 | na | na | na | neg | na |
| Pharynx swab | neg | neg | neg | na | na | na | na | na | na | na |
| Rectal swab | neg | neg | neg | na | na | na | na | na | na | na |
| Kidney | neg | 3e1 | 9.1e1 | na | 1.8e2 | na | na | na | 4.5e2 | na |
| Spleen | neg | 1.1e3 | 2.4e1 | na | neg | na | na | na | neg | na |

**Supplementary Table 6:**

Summary of tested specimens from the Netherlands

| **Country** | **Province** | **Species** | **Min year** | **Max year** | **# of specimens** |
| --- | --- | --- | --- | --- | --- |
| Netherlands | North Brabant | *P. pipistrellus* | 2018 | 2020 | 36 |
| Netherlands | South-Holland | *P. pipistrellus* | 2018 | 2019 | 16 |
| Netherlands | Groningen | *P. pipistrellus* | 2018 | 2018 | 10 |
| Netherlands | Flevoland | *P. pipistrellus* | 2018 | 2018 | 7 |
| Netherlands | Utrecht | *P. pipistrellus* | 2018 | 2018 | 5 |
| Netherlands | North-Holland | *P. pipistrellus* | 2018 | 2018 | 4 |
| Netherlands | Drenthe | *P. pipistrellus* | 2018 | 2018 | 3 |
| Netherlands | Limburg | *P. pipistrellus* | 2018 | 2018 | 2 |
| Netherlands | Gelderland | *P. pipistrellus* | 2018 | 2018 | 2 |
| Netherlands | Friesland | *P. pipistrellus* | 2018 | 2018 | 1 |

##

## Supplementary Figures


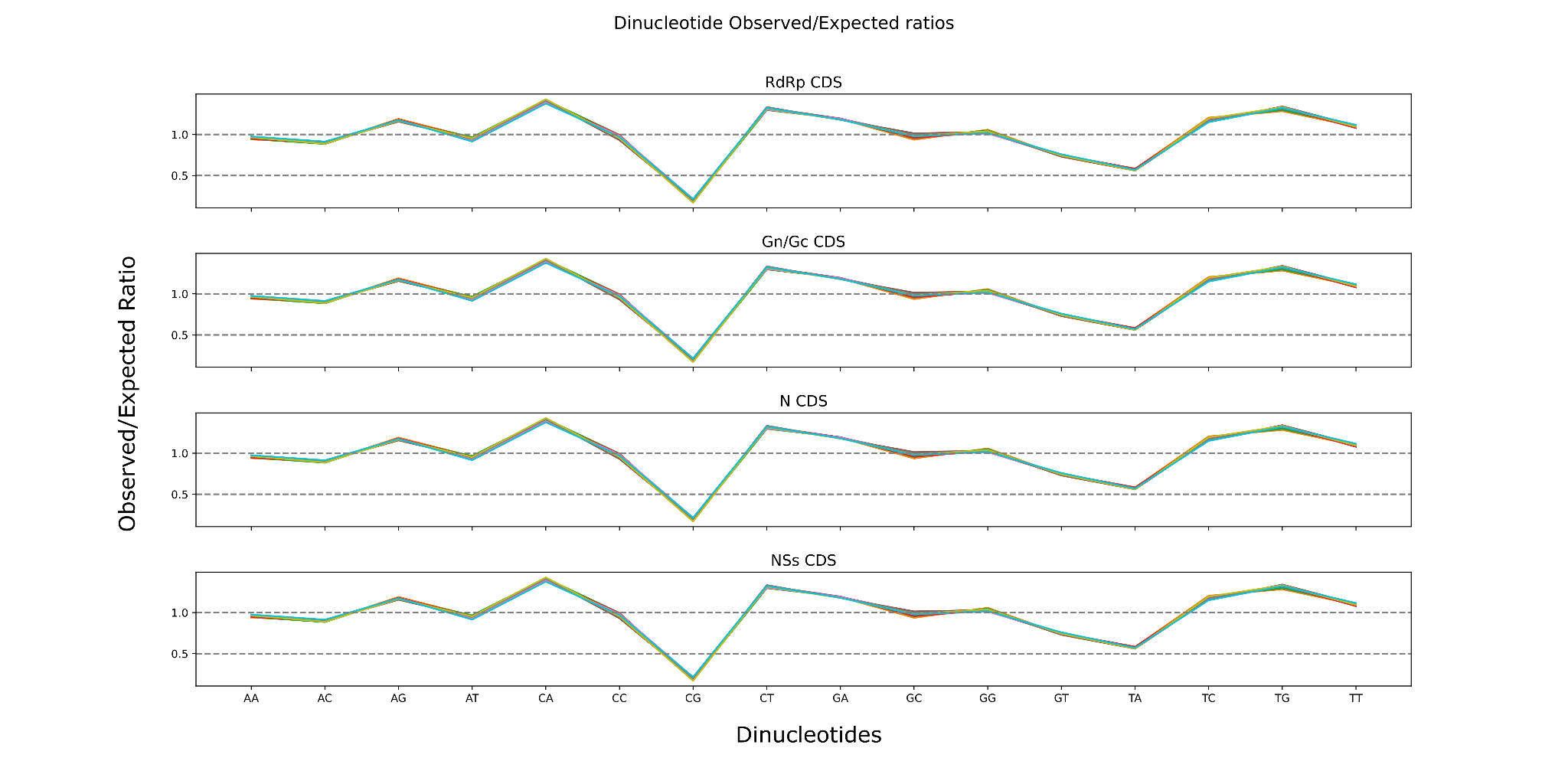


#### **Supplementary Figure 1:** Ratios of observed over expected dinucleotides in our recovered genomes, split by coding sequence. The ratios are presented across all ten genomes. However, due to the high degree of similarity among the genomes, the plotted lines overlap, making individual color labels indistinguishable and we therefore refrained from individual labels. The observed/expected ratio for each dinucleotide pair (XY) was calculated as the observed frequency of the dinucleotide (freq(XY)) divided by the product of the frequency of the individual nucleotides freq(X) and freq(Y) weighted by the length of the genome (freq(XY) x freq(X)^-1^freq(Y)^-1^ x genome_length), where unknown nucleotides (N’s) were excluded.


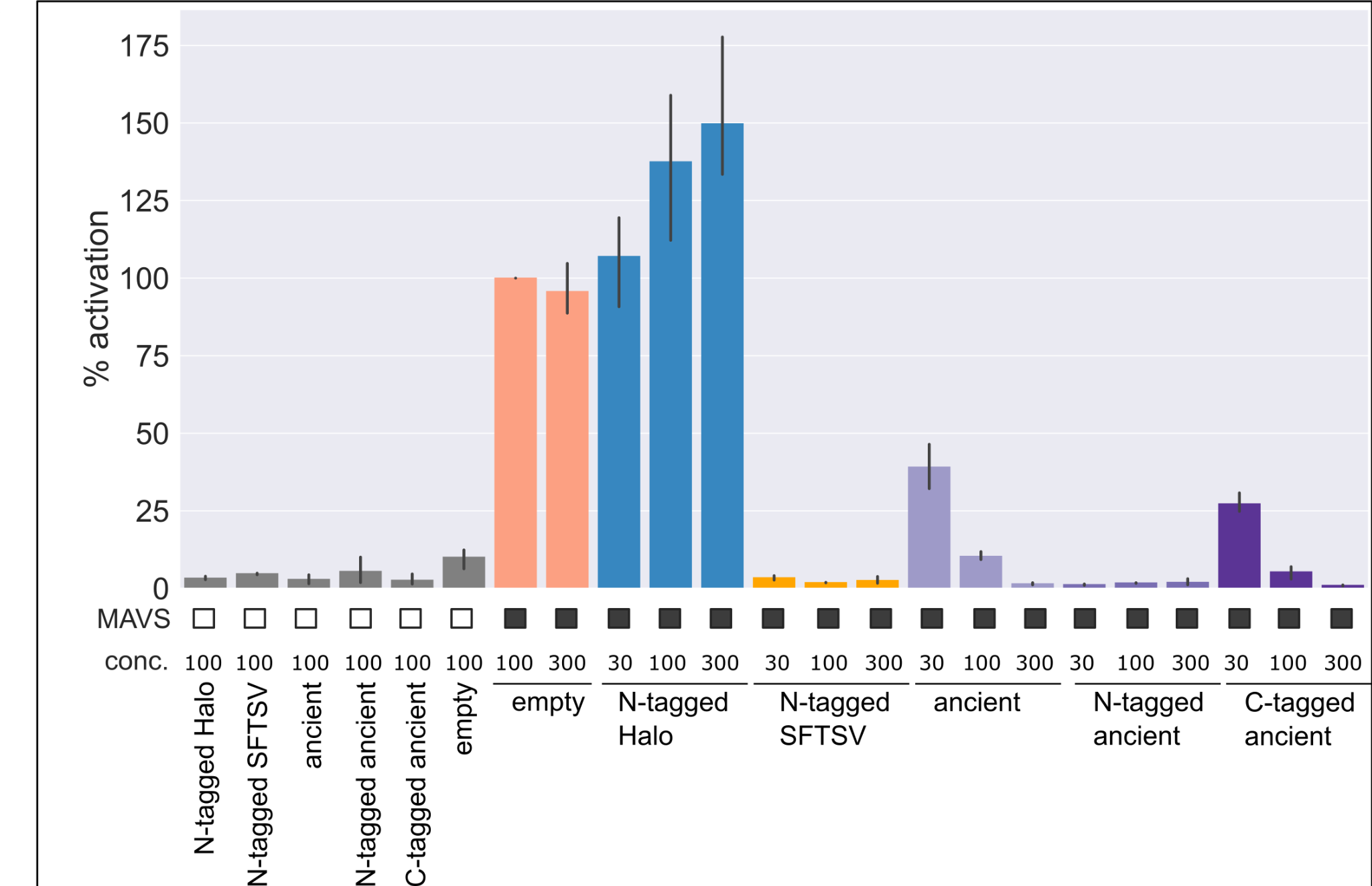


#### **Supplementary Figure 2:** Firefly luciferase assay to measure *Ifnb1* promoter activation via MAVS overexpression in human embryo kidney (HEK) 293T cells for the ancient Penzlin 1919 sequence. Black empty or filled squares indicate whether MAVS was overexpressed or not. Concentrations (conc.) of the respective plasmids are given in nanograms. Firefly luciferase activity was normalised to *Renilla* luciferase. The percentage of the activation of the empty vector (100 ng) is given for N-terminally 3×FLAG tagged HaloTag (N-tagged Halo) as additional negative control, N-terminally 3×FLAG tagged SFTSV NSs (N-tagged SFTSV), untagged Penzlin 1919 NSs (ancient), N-terminally 3×FLAG tagged Penzlin 1919 NSs (N-tagged ancient), C-terminally 3×FLAG tagged Penzlin 1919 NSs (C-tagged ancient). Error bars indicate estimated 95% confidence intervals.


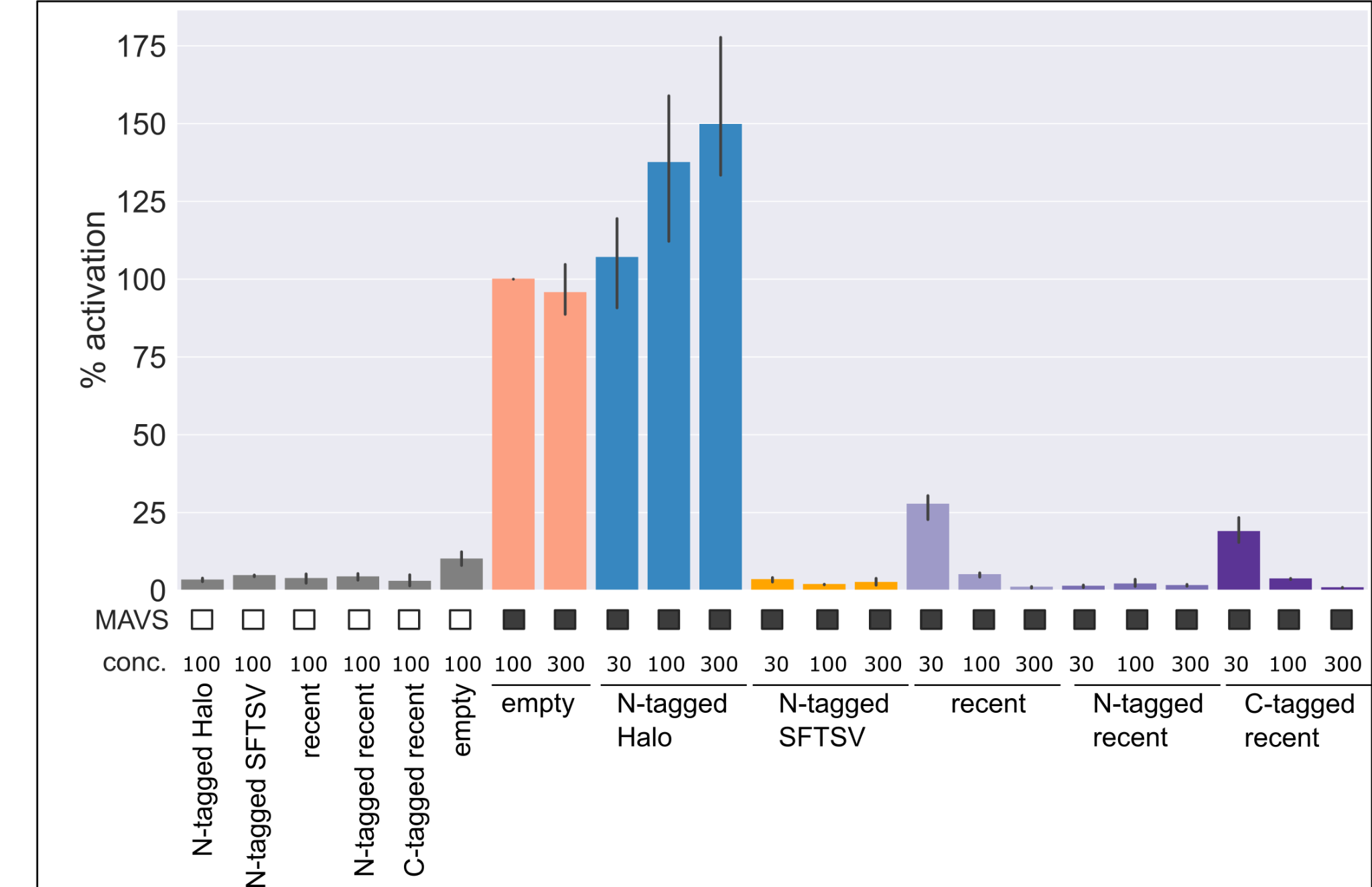


#### **Supplementary Figure 3:** Firefly luciferase assay to measure *Ifnb1* promoter activation via MAVS overexpression in HEK 293T cells for the recent Bad Lauterberg 2010 sequences. Black empty or filled squares indicate whether MAVS was overexpressed or not. Concentrations (conc.) of the respective plasmids are given in nanograms. Firefly luciferase activity was normalised to *Renilla* luciferase. The percentage of the activation of the empty vector (100 ng) is given for N-terminally 3×FLAG tagged HaloTag (N-tagged Halo) as additional negative control, N-terminally 3×FLAG tagged SFTSV NSs (N-tagged SFTSV), untagged Bad Lauterberg 2010 NSs (recent), N-terminally 3×FLAG tagged Bad Lauterberg 2010 NSs (N-tagged recent), C-terminally 3×FLAG tagged Bad Lauterberg 2010 NSs (C-tagged recent). Error bars indicate estimated 95% confidence intervals.

####

#### **
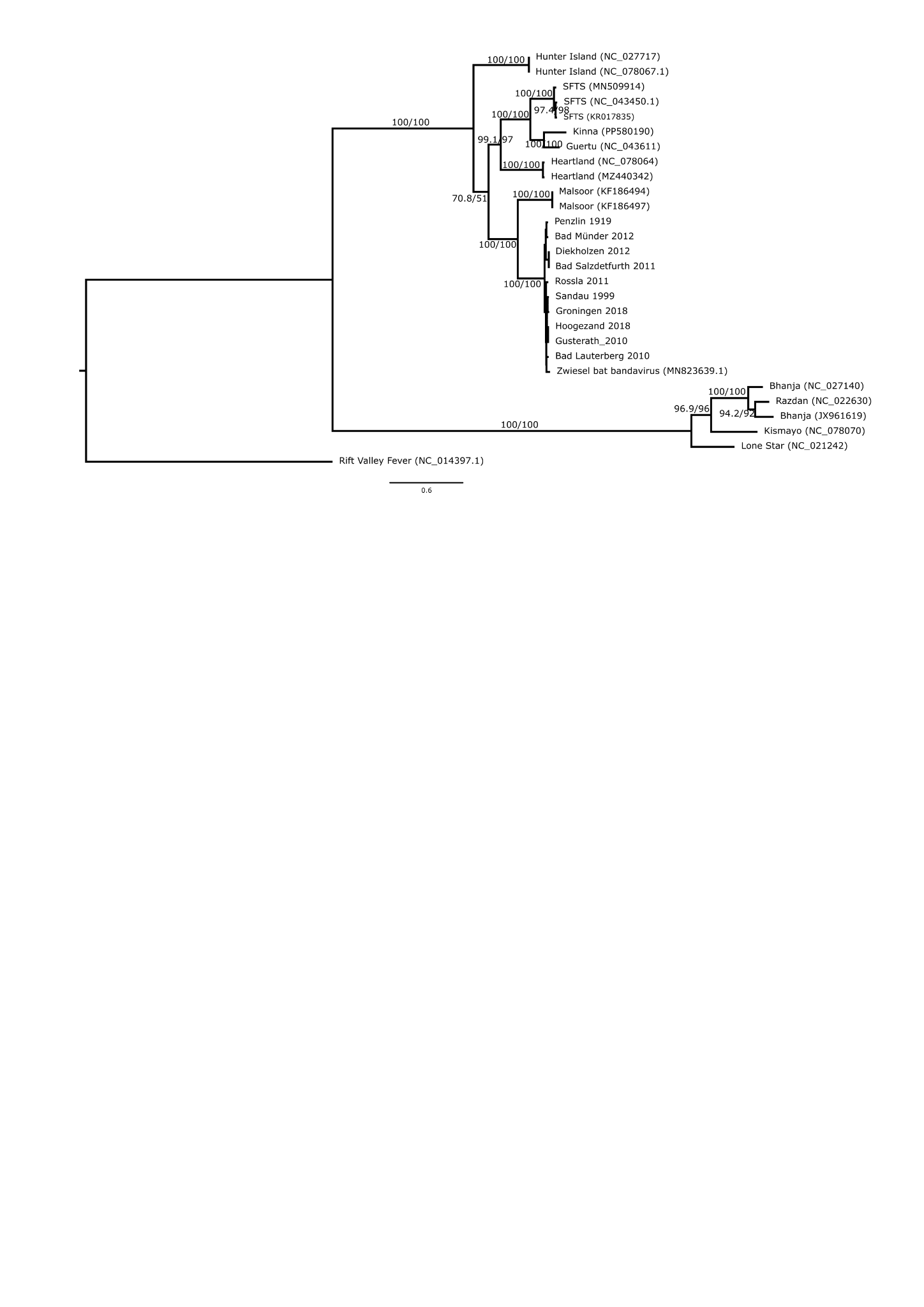
Supplementary Figure 4:** Phylogenetic relationships of L segment sequences of strains from the *Bandavirus* genus. The maximum likelihood tree was computed using IQ-TREE 2.3.6[^58^](https://app.readcube.com/library/443150cc-d401-49f5-962c-0e23b69b2feb/all?uuid=6236329631049148&item_ids=443150cc-d401-49f5-962c-0e23b69b2feb:544f3f87-615b-49e8-827b-a90faccd9765). Rift Valley Fever Virus (NC_014397.1) was used as an outgroup for rooting. Bootstrap support values were computed using UFBoot[^59^](https://app.readcube.com/library/443150cc-d401-49f5-962c-0e23b69b2feb/all?uuid=5395557838468121&item_ids=443150cc-d401-49f5-962c-0e23b69b2feb:6ba3d504-d820-4f8a-be02-1856679df110) and SH-aLRT[^60^](https://app.readcube.com/library/443150cc-d401-49f5-962c-0e23b69b2feb/all?uuid=15942468367915286&item_ids=443150cc-d401-49f5-962c-0e23b69b2feb:37ef22a7-e625-404c-a316-5444b29d5d29) and are both shown (in that order, slash-separated) on branches. Within the ZbbV clade no bootstrap support values are shown.

####
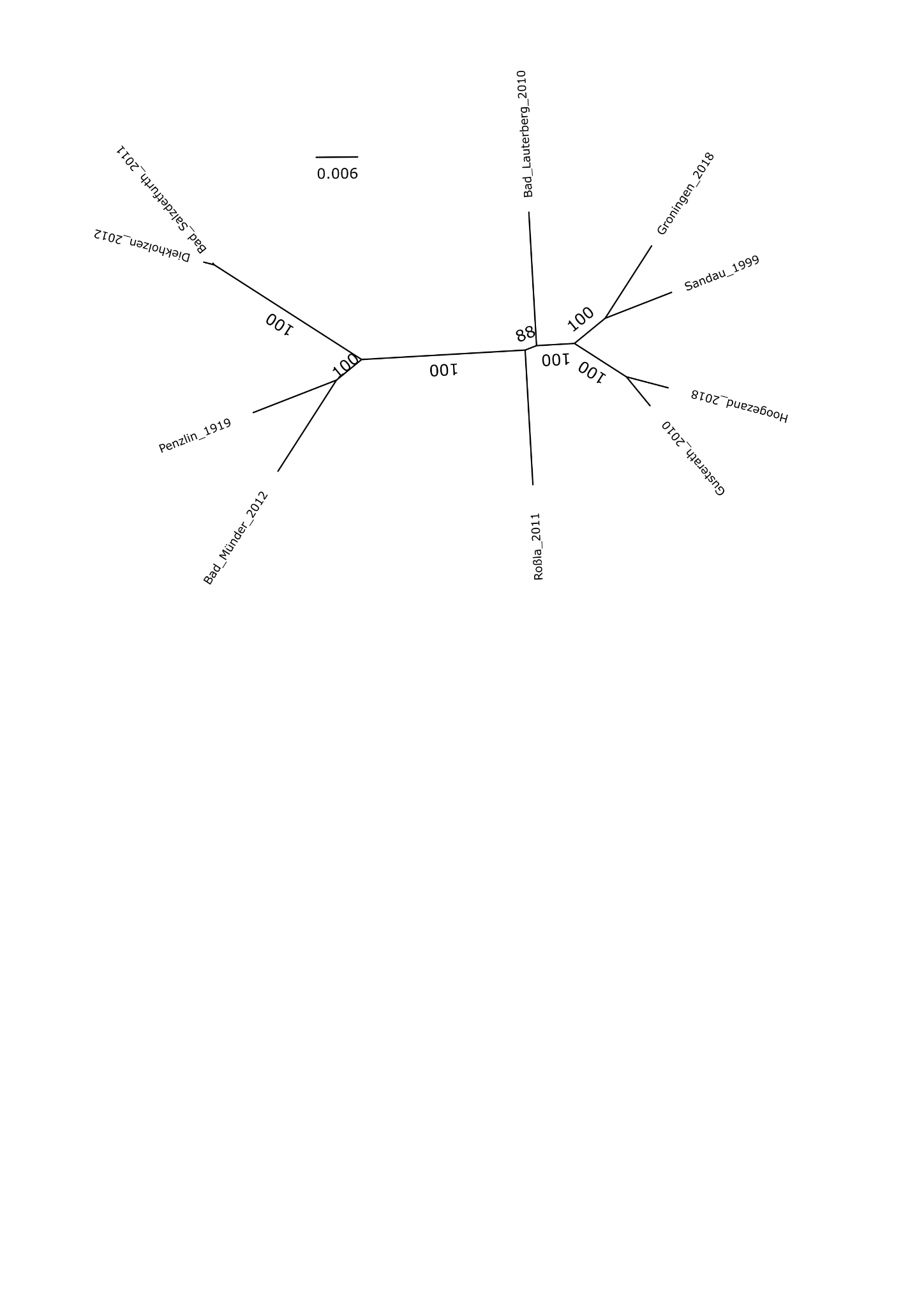
**Supplementary Figure 5:** Unrooted maximum likelihood tree of the RdRp CDS of our recovered genomes. Sequence alignment was performed with MUSCLE 5.1[^57^](https://app.readcube.com/library/443150cc-d401-49f5-962c-0e23b69b2feb/all?uuid=15545028787648918&item_ids=443150cc-d401-49f5-962c-0e23b69b2feb:78b415ea-cd3a-4970-afef-9cdc86a333d8), followed by ML tree construction in IQ-TREE 2.3.6[^58^](https://app.readcube.com/library/443150cc-d401-49f5-962c-0e23b69b2feb/all?uuid=25735785187087423&item_ids=443150cc-d401-49f5-962c-0e23b69b2feb:544f3f87-615b-49e8-827b-a90faccd9765), which included a comprehensive model search and bootstrap calculation with UFBoot[^59^](https://app.readcube.com/library/443150cc-d401-49f5-962c-0e23b69b2feb/all?uuid=9896455606124467&item_ids=443150cc-d401-49f5-962c-0e23b69b2feb:6ba3d504-d820-4f8a-be02-1856679df110) with support values shown on the branches.

#### **
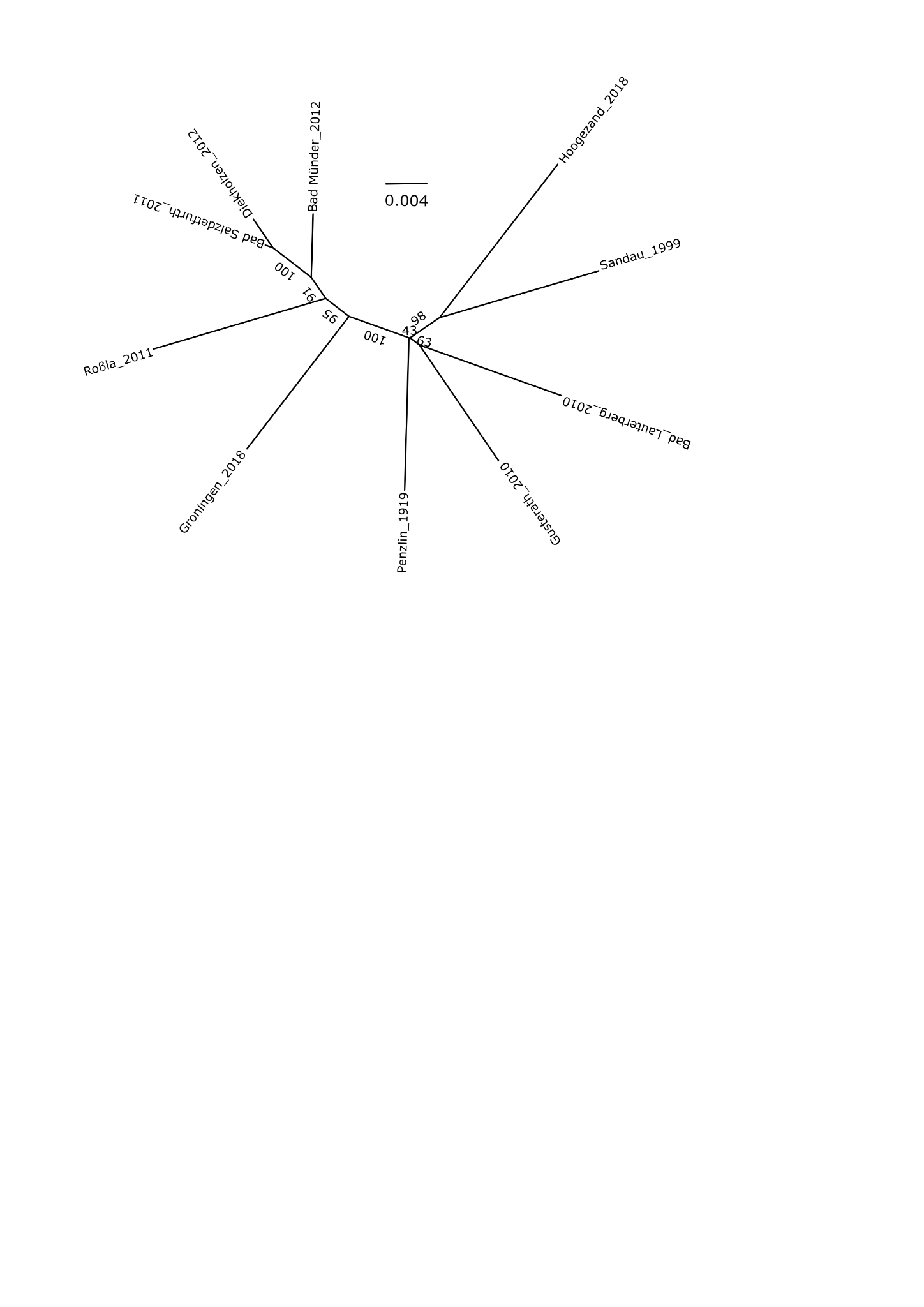
**

#### **Supplementary Figure 6:** Unrooted maximum likelihood tree of the Gn/Gc CDS of our recovered genomes. Sequence alignment was performed with MUSCLE 5.1[^57^](https://app.readcube.com/library/443150cc-d401-49f5-962c-0e23b69b2feb/all?uuid=7491648534672327&item_ids=443150cc-d401-49f5-962c-0e23b69b2feb:78b415ea-cd3a-4970-afef-9cdc86a333d8), followed by ML tree construction in IQ-TREE 2.3.6[^58^](https://app.readcube.com/library/443150cc-d401-49f5-962c-0e23b69b2feb/all?uuid=4247097200976573&item_ids=443150cc-d401-49f5-962c-0e23b69b2feb:544f3f87-615b-49e8-827b-a90faccd9765), which included a comprehensive model search and bootstrap calculation with UFBoot[^59^](https://app.readcube.com/library/443150cc-d401-49f5-962c-0e23b69b2feb/all?uuid=951732829058921&item_ids=443150cc-d401-49f5-962c-0e23b69b2feb:6ba3d504-d820-4f8a-be02-1856679df110) with support values shown on the branches.

####
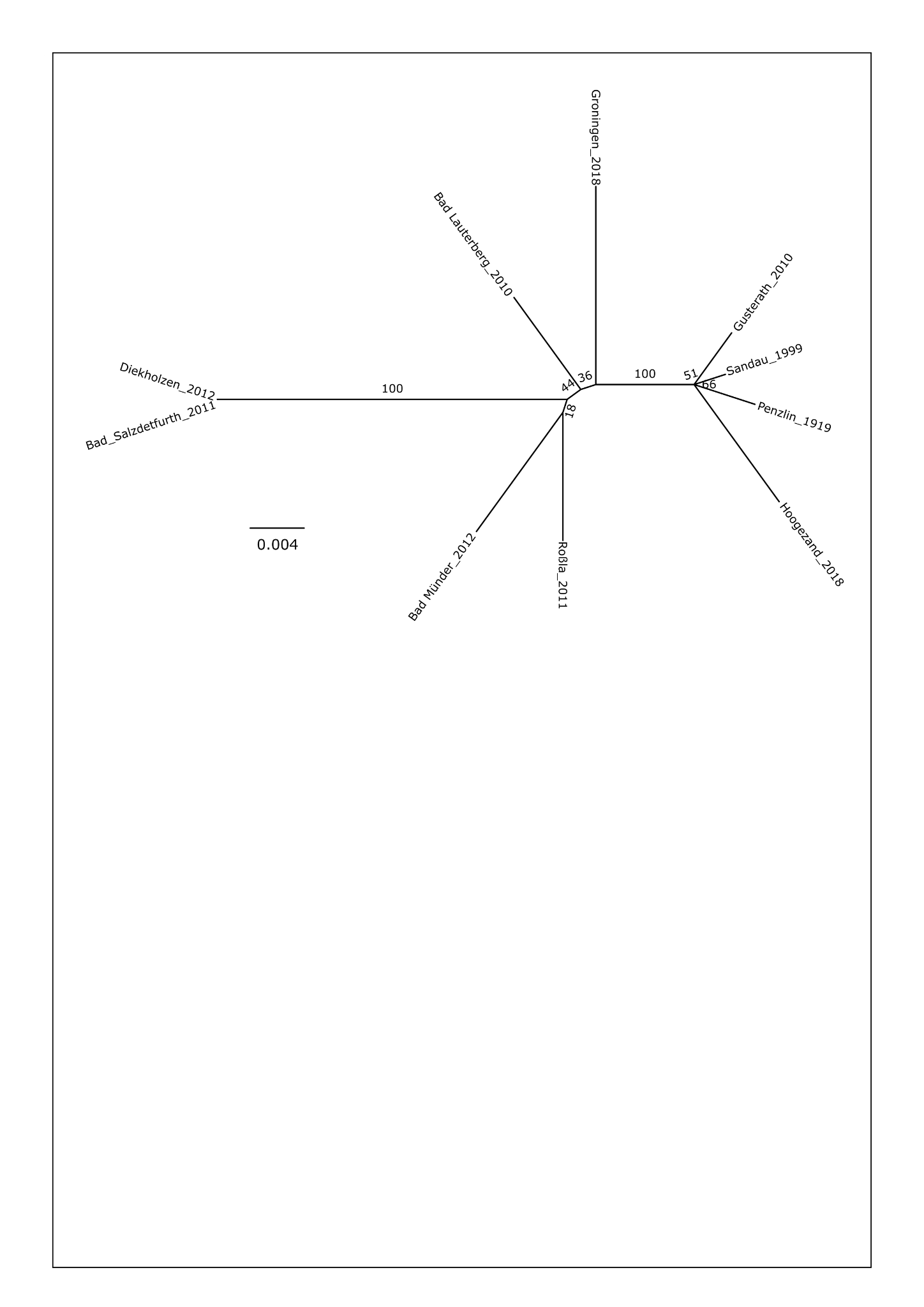


#### **Supplementary Figure 7:** Unrooted maximum likelihood tree of the NSs CDS of our recovered genomes. Sequence alignment was performed with MUSCLE 5.1[^57^](https://app.readcube.com/library/443150cc-d401-49f5-962c-0e23b69b2feb/all?uuid=12493639408370438&item_ids=443150cc-d401-49f5-962c-0e23b69b2feb:78b415ea-cd3a-4970-afef-9cdc86a333d8), followed by ML tree construction in IQ-TREE 2.3.6[^58^](https://app.readcube.com/library/443150cc-d401-49f5-962c-0e23b69b2feb/all?uuid=8571486522776308&item_ids=443150cc-d401-49f5-962c-0e23b69b2feb:544f3f87-615b-49e8-827b-a90faccd9765), which included a comprehensive model search and bootstrap calculation with UFBoot[^59^](https://app.readcube.com/library/443150cc-d401-49f5-962c-0e23b69b2feb/all?uuid=2650552602781534&item_ids=443150cc-d401-49f5-962c-0e23b69b2feb:6ba3d504-d820-4f8a-be02-1856679df110) support values shown on the branches.

####
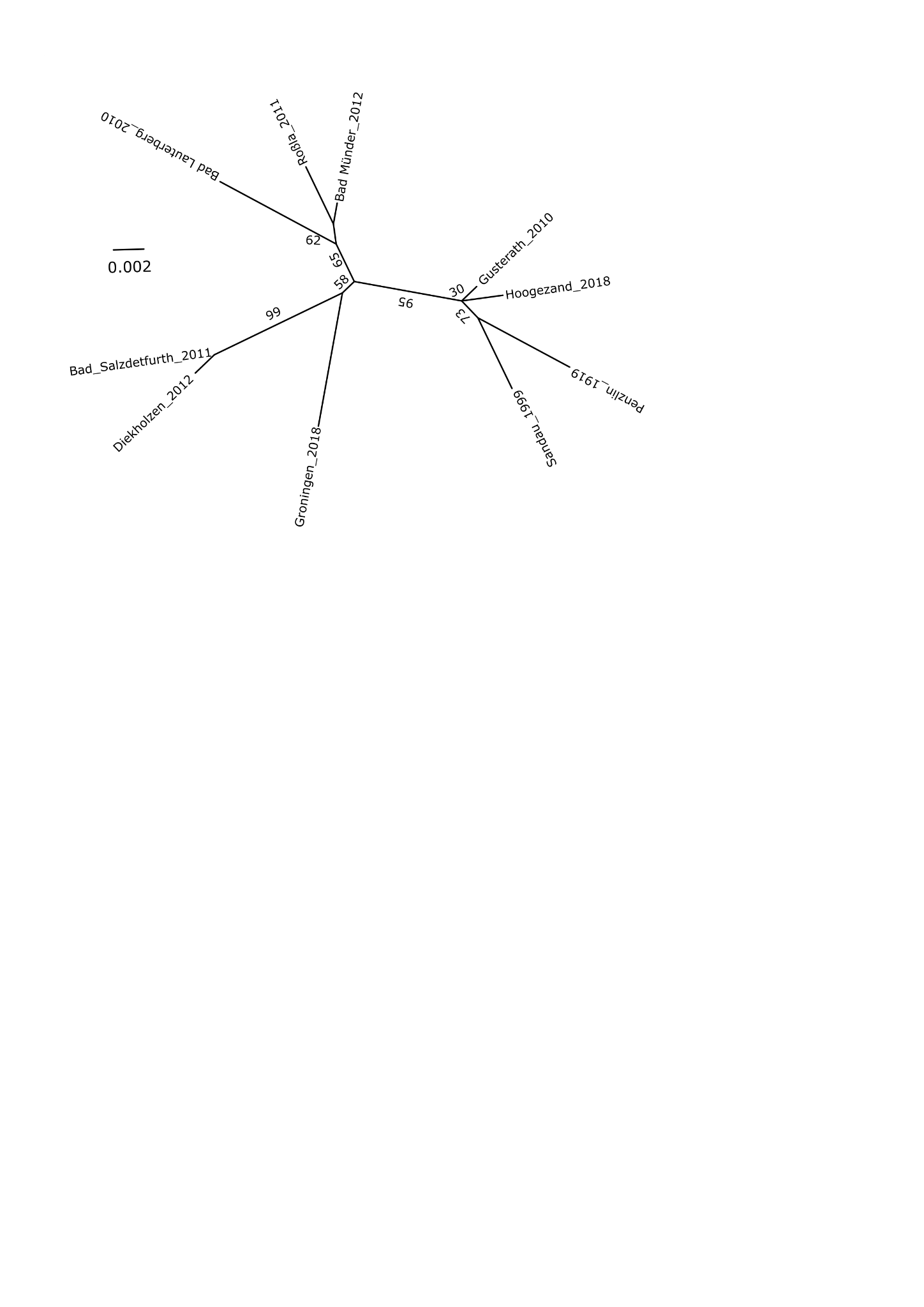


#### **Supplementary Figure 8:** Unrooted maximum likelihood tree of the N CDS of our recovered genomes. Sequence alignment was performed with MUSCLE 5.1[^57^](https://app.readcube.com/library/443150cc-d401-49f5-962c-0e23b69b2feb/all?uuid=3738553858278095&item_ids=443150cc-d401-49f5-962c-0e23b69b2feb:78b415ea-cd3a-4970-afef-9cdc86a333d8), followed by ML tree construction in IQ-TREE 2.3.6[^58^](https://app.readcube.com/library/443150cc-d401-49f5-962c-0e23b69b2feb/all?uuid=4807398503868521&item_ids=443150cc-d401-49f5-962c-0e23b69b2feb:544f3f87-615b-49e8-827b-a90faccd9765), which included a comprehensive model search and bootstrap calculation with UFBoot[^59^](https://app.readcube.com/library/443150cc-d401-49f5-962c-0e23b69b2feb/all?uuid=05345303366859211&item_ids=443150cc-d401-49f5-962c-0e23b69b2feb:6ba3d504-d820-4f8a-be02-1856679df110) support values shown on the branches.

#### **
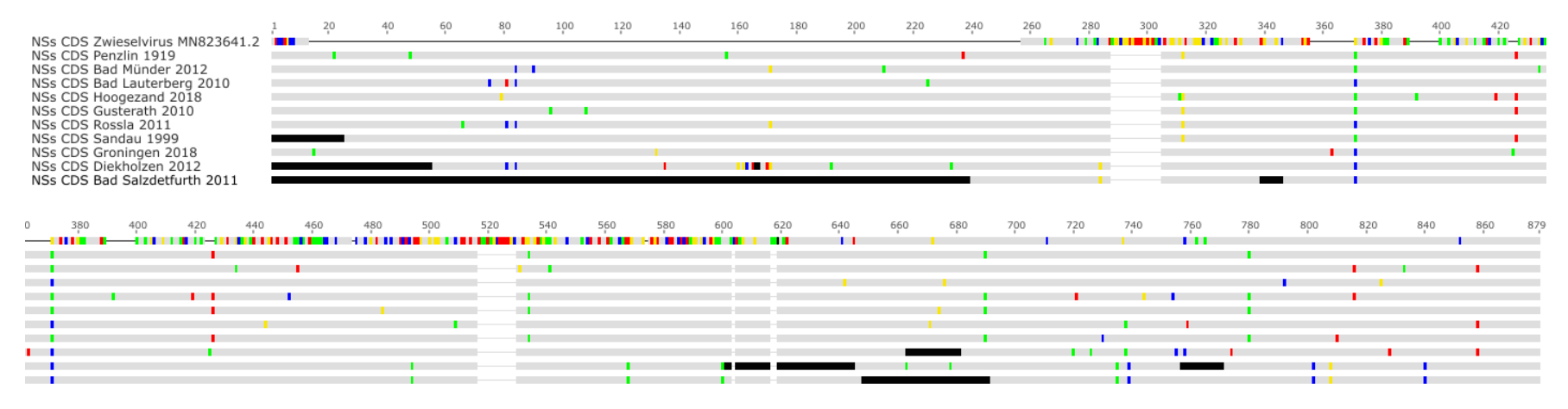
**

#### **Supplementary Figure 9:**

Sequence alignment (MUSCLE 5.1[^57^](https://app.readcube.com/library/443150cc-d401-49f5-962c-0e23b69b2feb/all?uuid=16713587823443588&item_ids=443150cc-d401-49f5-962c-0e23b69b2feb:78b415ea-cd3a-4970-afef-9cdc86a333d8)) of the NSs CDS of our recovered genomes and Zwiesel virus (MN823641.2). The alignment is visualized in Geneious Prime® 2024.0.7, highlighting differences to the overall consensus sequence in colour, black bars indicate missing sequence information. Zwiesel virus has a 606 nucleotide NSs CDS, whereas our genomes span 846 nucleotides, consistent with the 881 nucleotide length observed in the related SFTS virus NSs CDS[^76^](https://app.readcube.com/library/443150cc-d401-49f5-962c-0e23b69b2feb/all?uuid=5704898145550675&item_ids=443150cc-d401-49f5-962c-0e23b69b2feb:20db3cad-964e-47cd-a70e-3d047f66524f). The first ~340 nucleotides of the Zwiesel virus sequence show no alignment to any of our 10 genomes, while the remaining ~260 nucleotides align with our recovered genomes.

**
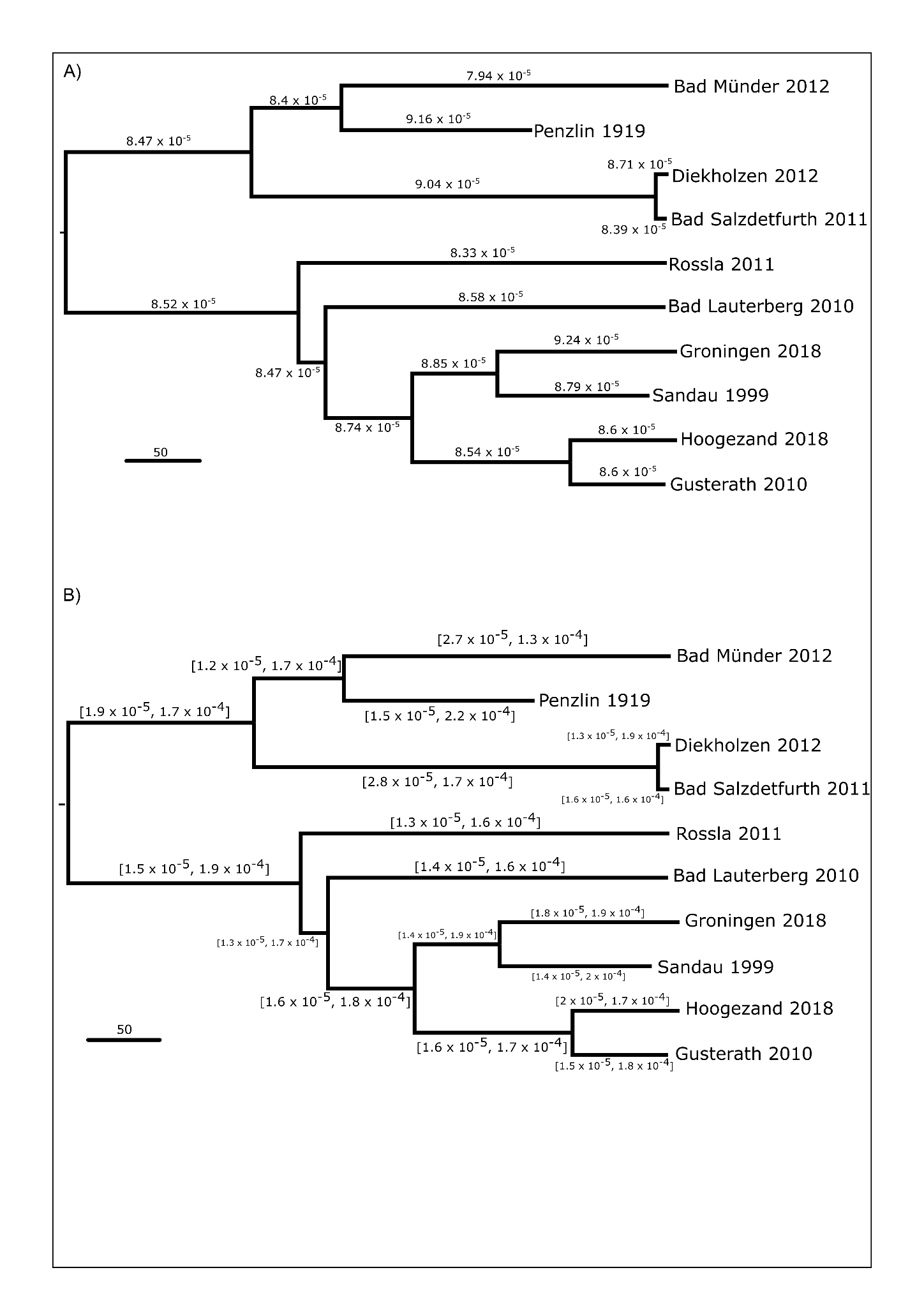
**

**Supplementary Figure 10:**Maximum clade credibility (MCC) tree showing the dated coalescent phylogeny estimated under a relaxed molecular clock model in BEAST v1.10.4[^64^](https://app.readcube.com/library/443150cc-d401-49f5-962c-0e23b69b2feb/all?uuid=505996680861859&item_ids=443150cc-d401-49f5-962c-0e23b69b2feb:91e9cd47-44e9-4354-a2cc-e3f9c4288efa). **A)** MCC tree with branch annotations indicating the median of the posterior substitution rate estimates. **B)** The same tree with branch annotations showing the 95% highest posterior density intervals of the sampled substitution rates.


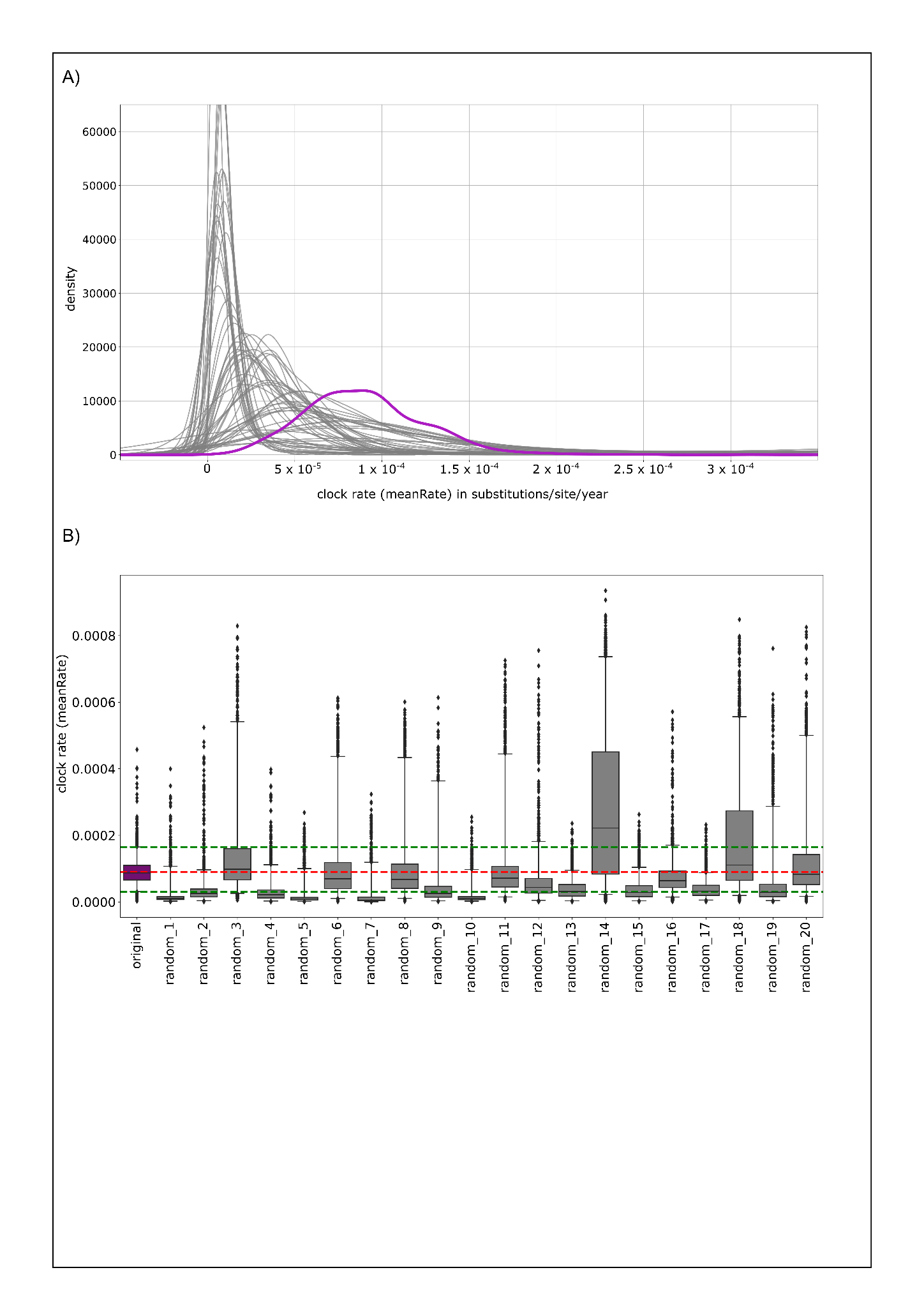


#### **Supplementary Figure 11:** Date-randomization test showing the average of the posterior rate estimates (meanRate) across the branches of the respective phylogenetic trees inferred with correct versus randomized tip dates. **A)** Posterior distributions of the average rate (meanRate) for 53 datasets with randomized sampling dates (grey), with the posterior from the original dataset shown in purple. **B)** Boxplot summarizing the average rate (meanRate) across 20 randomized datasets (grey). Whiskers indicate the 95th percentile range. The original dataset is shown in purple, with its mean (red) and 5th and 95th percentiles (green).
